# The 3D Printing of Flexible Materials: Technologies, Materials, and Challenges

**DOI:** 10.3390/ma18235428

**Published:** 2025-12-02

**Authors:** Suyun Li, Zengqin Shi, Yixuan Wang, Wenqing Wang, Rujie He

**Affiliations:** Institute of Advanced Structure Technology, Beijing Institute of Technology, Beijing 100081, China; lsy_2010fly@163.com (S.L.); skdnanozqs@163.com (Z.S.); 3220232673@bit.edu.cn (Y.W.); wangwq0628@163.com (W.W.)

**Keywords:** flexible materials, 3D printing, additive manufacturing

## Abstract

Due to their unique functional properties, such as deformability, bendability, stretchability, and even biocompatibility, sensing, or actuation, flexible materials have become an indispensable and crucial component in electronic systems such as wearable electronic devices and soft robots. Facing the complex demands of various application scenarios, 3D printing technology can be utilized to customize the preparation of various flexible materials into desired shapes. However, compared to rigid materials, flexible materials still face printing issues such as pore defects and weak interlayer bonding during the 3D printing process. Therefore, this paper focuses on analyzing the key bottleneck issues and technical challenges currently existing in flexible material 3D printing technology, and provides an overview of the progress in preparing flexible materials using 3D printing technologies, such as Material Extrusion and Vat Polymerization. Finally, it looks forward to the technical challenges and future development of 3D printing with flexible materials.

## 1. Introduction

The core principle of additive manufacturing (also known as 3D printing) technology is to directly construct three-dimensional solid structures by precisely stacking materials layer by layer based on digital models [1,2]. Compared to traditional subtractive manufacturing (such as cutting and milling) and isometric manufacturing (such as casting and forging), the most significant advantages of 3D printing technology lie in its mold-free, high-degree-of-freedom design, integrated molding of complex structures, and highly customized capabilities. Technologies such as fused deposition modeling (FDM), stereolithography (including SLA/DLP), and selective laser sintering (SLS) have been successfully applied in various fields such as aerospace and biomedical, achieving a leap from concept verification to direct manufacturing of functional components [3,4]. In the development of 3D printing technology, the printing demand for flexible materials has become increasingly prominent, and it has gradually become one of the key directions for expanding the application fields of 3D printing technology [5,6,7,8,9,10].

**Figure 1:**
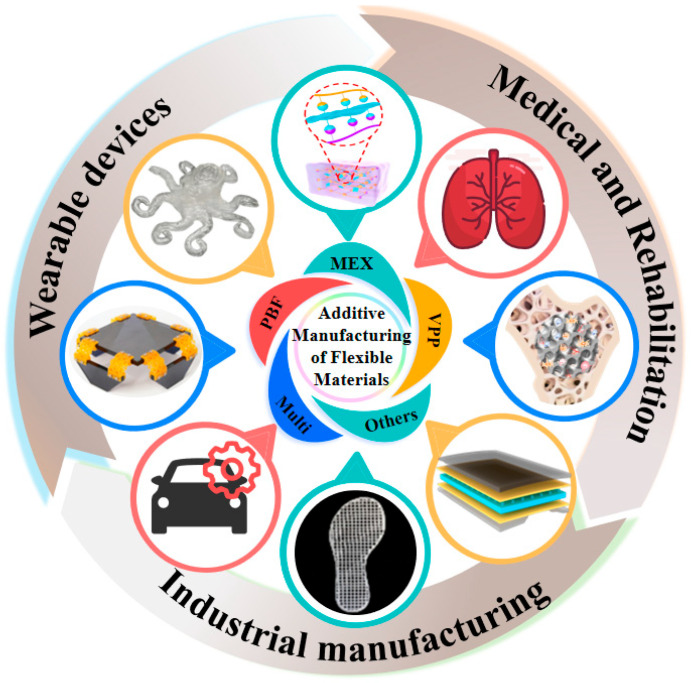
Applications and development overview of flexible materials based on additive manufacturing. Sources: [15,17,19,20,21].

Flexible materials endow printed products with unique functional properties such as deformability, bendability, stretchability, and even biocompatibility, sensing, or actuation [8,10]. However, traditional manufacturing methods, such as mold forming, often incur high costs, require long cycles, and exhibit poor flexibility when manufacturing flexible components with complex geometries. On the other hand, 3D printing technology provides unprecedented opportunities for the personalized, complex, and functionally integrated manufacturing of flexible structures, becoming a key technology for realizing multi-scenario applications of flexible materials. For instance, 3D printing can be used to fabricate soft tissue scaffolds with high conformity to human tissue compliance [11] and biocompatibility, soft robots that adapt to complex unstructured environments, and wearable electronic device substrates that comfortably fit the human body, as shown in Figure 1 [12,13,14,15,16,17,18,19,20,21].

The research on 3D printed flexible materials not only drives the transformation and innovation of the manufacturing industry, but also opens up new avenues for personalized customization and functional product development. Despite its promising prospects, successfully applying flexible materials to 3D printing and achieving high-performance, high-precision, and high-reliability functional component manufacturing faces more severe material innovation and process challenges than printing rigid materials [9,22,23]. This is due to the fact that flexible materials (especially elastomers and hydrogels) typically exhibit characteristics such as high viscosity, low modulus, ease of deformation, and complex curing/crosslinking mechanisms. These characteristics result in poor compatibility with existing mainstream 3D printing processes, such as Fused Deposition Modeling (FDM) relying on melt flow, Stereolithography (SLA) and Digital Light Processing (DLP) relying on light curing, and Selective Laser Sintering (SLS) relying on powder melting [8,10,22,24,25,26,27]. Developing novel flexible printing materials that possess both excellent printability (such as suitable rheological properties, photosensitivity, and powder characteristics) and targeted functionalities (such as high elasticity, high toughness, conductivity, and bioactivity) and establishing a mapping relationship between material properties and printing processes are key challenges that urgently need to be addressed [9,10,28,29,30,31,32,33]. However, the rapid development of personalized medicine, soft robots, and flexible electronics has led to a surge in demand for flexible devices with complex geometries, heterogeneous material distributions, and embedded functionalities (such as sensing and actuation), continuously driving the development of flexible material 3D printing technology towards higher performance, higher precision, and greater intelligence [34,35,36,37,38].

Therefore, this article systematically reviews the current status of research on 3D printing technology for flexible materials, summarizing the types of flexible materials that can be 3D printed and their research status. It then analyzes the key bottleneck issues and technical challenges currently existing in 3D printing technology for flexible materials, and discusses the innovation of 3D printing materials for flexible materials, performance regulation, and process challenges, as well as the future development direction of the technology. This review aims to focus on the theme of “3D printing technology for flexible materials”, concentrating on the innovative breakthroughs in material systems and the core challenges faced by printing processes, for a comprehensive and in-depth discussion and commentary.

## 2. Advances in 3D Printing of Flexible Materials

Flexible materials, typically referring to substances with a low Young’s modulus, high elongation at break, good elastic recovery properties, or viscoelasticity, encompass thermoplastic elastomers (TPE, TPU), silicone rubber, hydrogels, organic gels, and various composite materials. With the increasing demand for complex structural components in various fields, the research on flexible materials has also been gaining popularity. The combination of 3DP technology and flexible materials has made the fabrication of various flexible complex structures a reality. With the iterative upgrading of 3D printing technology and the continuous enrichment of material systems, 3D printing is steadily advancing from prototype manufacturing to end-product manufacturing, and from small-batch customization to large-scale production, demonstrating great potential to reshape the future manufacturing landscape.

### 2.1. 3D Printing Technologies

The 3D printing technologies commonly used for preparing flexible materials mainly include Material Extrusion (MEX) and Vat Polymerization (VPP), in addition to Powder Bed Fusion (PBF), Binder Jetting (BJT), and other preparation methods that combine other technologies with 3D printing technology. As shown in Table 1, the currently mainstream 3D printing technologies for preparing flexible materials and their advantages and disadvantages are presented.

#### 2.1.1. Material Extrusion

MEX 3D printed flexible materials have significant advantages and limitations. Their core advantage lies in their wide material compatibility, especially their good adaptability to thermoplastic elastomers (such as TPU, TPE). These materials can achieve a fracture elongation rate of 50–500% through melt extrusion, meeting the needs of most flexible applications [65]. Among them, fused deposition modeling (FDM) and direct ink writing (DIW) are two commonly used printing methods. FDM involves heating various thermoplastic polymers to induce melting and extrusion, and then forming complex structures layer by layer. DIW printing technology is a printing method that uses functional inks to extrude and form layer by layer at room temperature. Currently, MEX technology has been successfully applied in cutting-edge fields such as tissue engineering and soft robotics. Furthermore, the multi-material printing technology developed in recent years has further expanded its versatility, enabling co-extrusion, mixing, and material switching. However, its further development is still limited by challenges such as insufficient bonding strength at the multi-material interface and optimization of process parameters [65,66].

**Figure 2:**
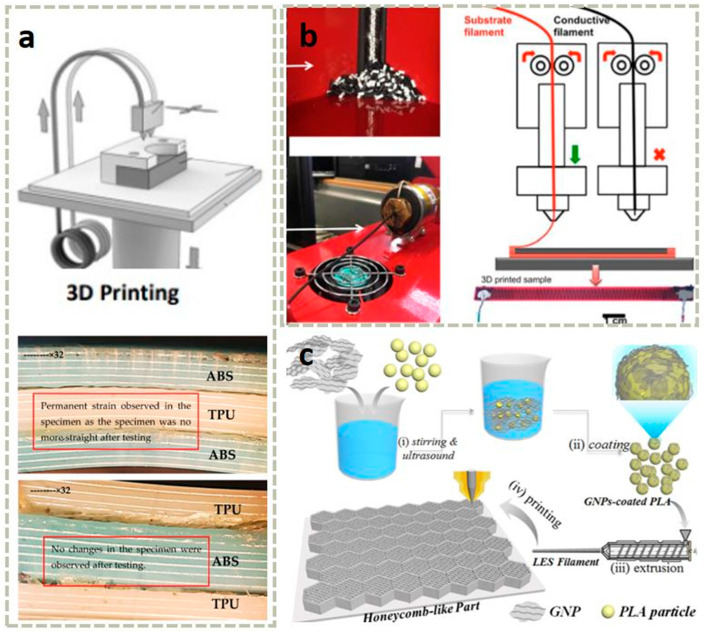
(**a**) FDM printing technology for multi-material flexible composite materials; (**b**) FDM printing technology for polymerization processes; (**c**) FDM printing technology for printable flexible materials with functional fillers. Sources: [85,86,87].

FDM is a 3D printing technology based on hot-melt extrusion. Its principle involves heating a thermoplastic filament to a temperature slightly above its melting point using a heated nozzle, causing it to melt into a semi-fluid state. Under computer control, the nozzle then moves along the X–Y plane and extrudes the molten material according to the model’s slice contour, depositing it layer by layer on a work platform to ultimately build up a three-dimensional solid. Through FDM technology, filaments of thermoplastic materials such as Poly Lactic Acid (PLA) [67], Polydimethylsiloxane (PDMS) [68], Polyurethane (TPU) [69,70,71,72,73], Polycaprolactone (PCL) [74], Acrylonitrile Butadiene Styrene (ABS) [75,76,77], polycarbonate [78,79,80,81,82,83], and polyphenylsulfone [84] can be transformed into three-dimensional structures. Furthermore, as shown in Figure 2a, through multi-material composite printing, the bending strength of the material increases from approximately 6.8 MPa to 13 MPa, representing a nearly 92% increase in bending strength [85]. Meanwhile, by co-printing graphene-based polylactic acid (PLA) and thermoplastic polyurethane (TPU), it is possible for the first time to 3D print highly stretchable and sensitive strain sensors based on graphene composite materials in strain sensors (as shown in Figure 2b) [86]. In addition, as shown in Figure 2c, by embedding functional fillers (such as nanoparticles, fibers, or conductive materials) into the wire matrix [87], the multifunctional applications of this technology in fields such as smart devices and biomedicine can be expanded.

For example, by combining PLA and PDMS, a highly flexible pressure sensor for monitoring health signals such as wrist pulse, swallowing, and vocalization can be prepared, demonstrating the practical application value of flexible materials [68]. Alternatively, the functional thermoplastic material PVDF can be printed via FDM to produce flexible sensors for motion capture in patients with Parkinson’s disease [88]. Additionally, continuous carbon fiber can be added to TPU to enhance the flexibility of the substrate material [72]. In addition, by flexibly combining FDM technology with other processes, precise manufacturing can be achieved. For instance, by combining FDM 3D printing with the standard pharmaceutical production process of hot-melt extrusion (HME), the linear relationship between quality and print volume is preserved, and the dosing accuracy range of three methacrylic acid polymers (Yudeli RL, RS, and E) and one cellulose-based material (hydroxypropyl cellulose, HPC SSL) is controlled between 91% and 95% [89].

**Figure 3:**
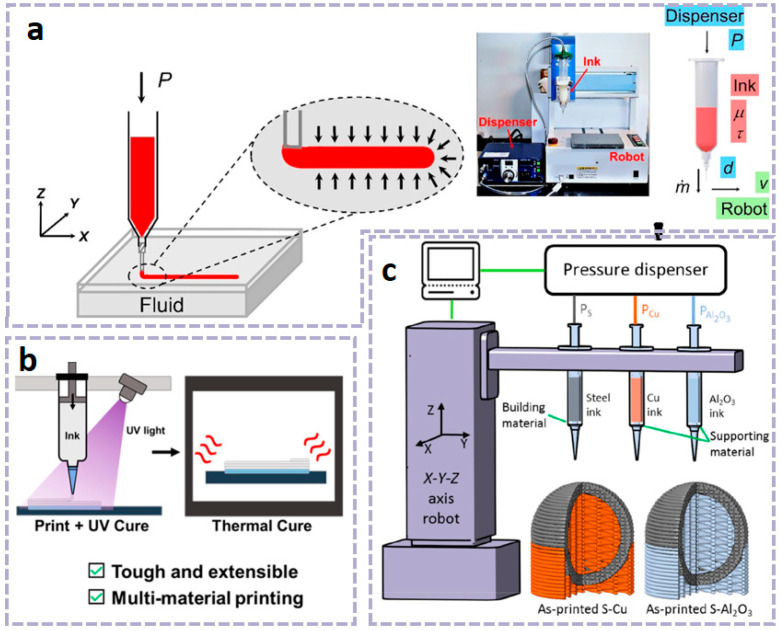
(**a**) DIW printing technology with fluid support; (**b**) DIW printing technology with UV curing capability; (**c**) DIW printing technology using multiple materials to achieve easily removable supports. Sources: [18,66,95].

As a commonly used material extrusion printing method, DIW technology is compatible with the widest range of materials, provided that the precursor ink is engineered to exhibit appropriate rheological properties [15,90]. At the same time, DIW technology can overcome the limitations of FDM in terms of temperature and material requirements. Various materials, including ceramics and polymer composites, have been selected as inks for DIW printing, which can be applied in areas such as electronic skin, wearable devices, flexible batteries, and biomedicine [91,92,93]. However, in order to obtain a stable structure, it is necessary to carefully adjust and optimize the rheological, dimensional, and thixotropic properties of the printing slurry based on parameters such as nozzle diameter, stroke spacing, and extrusion speed of the equipment [94]. To successfully develop water-based polyurethane ink suitable for DIW 3D printing, researchers achieved direct printing of complex, integral elastic structures at room temperature while maintaining the designed morphology by introducing cellulose nanofibrils (CNFs). Simultaneously, the introduction of solvent-induced rapid solidification (SIFS) technology not only enhanced the mechanical properties but also enabled room-temperature curing [21]. For different printing pastes (such as hydrogel, ceramic pastes, etc.), different adjustments and controls are required, and post-processing is targeted. However, due to the influence of surface tension and gravity, DIW has limitations in printing complex shapes such as arched structures and suspended structures, which may lead to material sagging or buckling deformation. Researchers have broken through the limitations of traditional DIW technology by using solid sugar powder as a support medium, achieving precise manufacturing of complex arch structures and suspension components [94]. As shown in Figure 3a, researchers have developed a simple method to fabricate planar microstructures composed of polysiloxane using commercially available liquid polysiloxane resin without altering its properties. Using a DIW printer, the curable liquid polysiloxane (with a viscosity range of 1–100 Pa·s) is formulated, and the liquid is immiscible with resins such as methanol, ethanol, and isopropanol. The contact angle (θ) of the dispensed polysiloxane on a Petri dish increases from 20° in air to 100° in methanol, ethanol, and isopropanol. The increase in contact angle allows the patterned polysiloxane structure to be maintained until curing, and the embedded liquid can be easily removed through evaporation. We refer to this method as EIW. The effects of curing time (τ) and nozzle speed (v) on the width of the printed filaments (w) were evaluated. EIW achieved a minimum width of 65 μm for the printed filaments. EIW enables direct writing of polysiloxane resin and holds promise for applications in the fabrication of microfluidic devices, flexible wearable devices [66].

Matthew et al. [18] proposed a method for printing commercial thermosetting polyurethane elastomers using a UV–DIW dual-curing approach. This hybrid dual-curing resin consists of photopolymerizable acrylate monomers for rapid shape fixation and thermosetting polyurethane monomers to provide customizable elastomeric mechanical properties. By tuning the composition of the acrylate and polyurethane networks, a wide range of mechanical properties, from soft elastomers (E~2 MPa) to rigid plastics (E~1 GPa), was achieved. Through atomic force microscopy studies of phase behavior and internetwork penetration, it was found that the dual-cured polymer possesses a two-phase microstructure with submicron-sized domains, and undergoes matrix inversion as the composition changes. The polyurethane elastomer can be printed via UV–DIW with an extremely low acrylate content (20 wt%) and exhibits excellent mechanical properties, including high elongation (>600%) and toughness (>10 MJ m^–3^). Research indicates that this method is capable of generating multi-material parts with different stiffness regions, suitable for applications such as pneumatic soft actuators, and exhibits excellent adhesion between adjacent regions and layers (as shown in Figure 3b).

Based on the characteristic that the supports printed from the same material become inseparable from the building structure after heat treatment, Xu et al. [95] developed a multi-material DIW method. This method involves creating removable supports, which are printed from low-melting-point metals or ceramics, to fabricate complex three-dimensional steel structures. The low-melting-point metals fully penetrate the porous steel structure, achieving a hybrid structure, while the ceramics provide brittle supports that are easy to remove. By characterizing dimensional shrinkage, surface roughness, filament porosity, electrical conductivity, and tensile properties, the study investigated the impact of support materials on the performance of steel structures. The hybrid structure improved the electrical conductivity of the steel structure by 400% and increased its mechanical stiffness by 34%. Alumina supports are physically and chemically stable during heat treatment and do not significantly contaminate the steel structure (as shown in Figure 3c).

In summary, compared to traditional processing methods, MEX, as one of the main 3D printing technologies for preparing flexible materials, has advantages such as easy operation, but still faces many problems. Based on the printing principle of MEX, it is not difficult to find that it faces issues such as low printing resolution, complex post-processing, and nozzle blockage.

#### 2.1.2. Vat Polymerization

VPP technology is a 3D printing technology based on light curing. It selectively irradiates liquid photosensitive resin with ultraviolet light or laser to trigger monomer polymerization, achieving layer-by-layer curing and molding. Its core advantages lie in high precision (micrometer to nanometer level) and the ability to manufacture complex structures, making it suitable for applications in micro nanoelectromechanical systems, biomedicine, optical devices, flexible sensing [5,6,96], and other fields. Traditional VPP is limited to a single material. In recent years, high-precision preparation at the micro–nano scale using multiple materials has been achieved through technologies such as dynamic fluid delivery and multi-ink tank switching [97].

**Figure 4:**
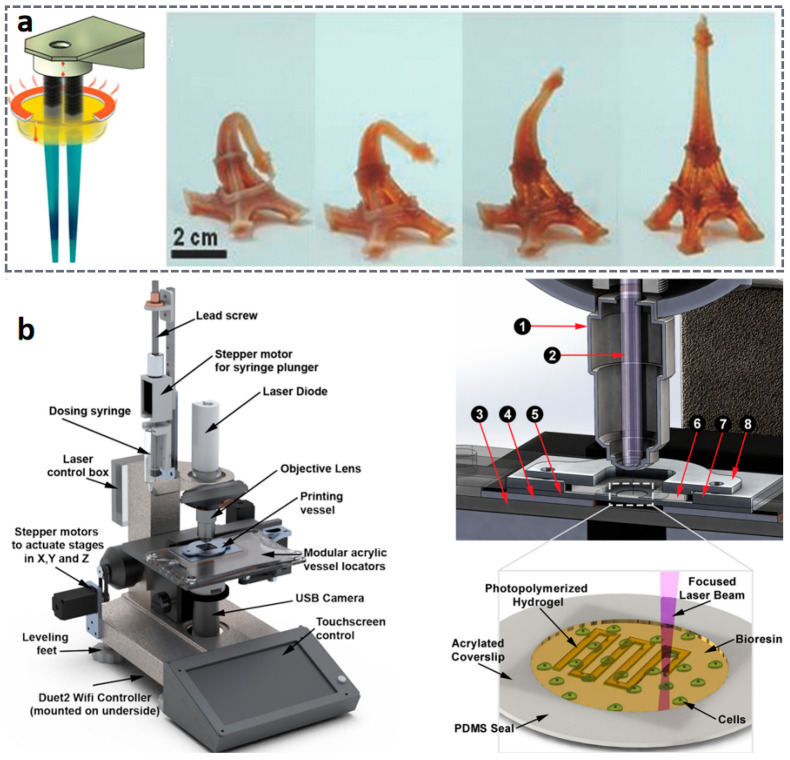
(**a**) SLA printing technology that can print non-liquid resins; (**b**) SLA bioprinting technology using customized visible light. Sources: [101,112].

As the earliest VPP technology, SLA primarily achieves solidification and molding of liquid resin through point-by-point scanning with an ultraviolet laser. Modern SLA systems, through system optimization, can achieve printing precision ranging from 25 to 100 μm, but the printing speed is relatively slow [98,99,100]. Researchers have long conducted studies on the preparation of flexible materials based on SLA technology. As shown in Figure 4a, Matt et al. successfully printed oligomer melts instead of liquid resin using SLA technology, preparing high-resolution three-dimensional shape memory structures, which were then used to construct flexible electronic devices [101]. In addition, SLA technology has been applied to the preparation of multifunctional hydrogels with complex microstructures [102,103,104,105,106], which can be utilized in biomedicine, tissue engineering, wearable devices, and more [107,108,109,110,111]. However, the use of SLA equipment to prepare hydrogel filaments with diameters less than 20 μm has received relatively little attention. To this end, Viray et al. [112] developed a customized visible light SLA bioprinting system named “MicroNC”. This system successfully prepared hydrogel scaffolds with clear linear structures (widths less than 8 μm) for the first time by using a novel visible light bioresin material (as shown in Figure 4b). Furthermore, to further enhance resin energy absorption during the SLA printing process, researchers have developed an SLA technology based on liquid crystal displays (LCDs), which has been applied in drug delivery, the fabrication of microfluidic devices, and piezoelectric materials [113]. However, during the three-dimensional (3D) compression analysis of different parts printed by the SLA process, key information regarding energy absorption remains limited. During SLA laser scanning, fillers may cause laser path deviation, affecting edge clarity.

**Figure 5:**
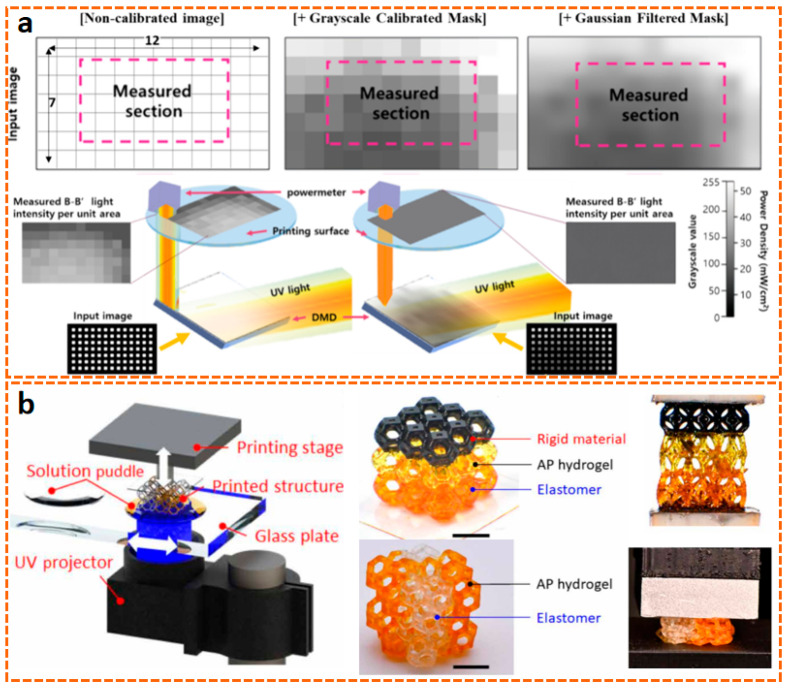
(**a**) DLP printing technology with improved layer-by-layer algorithms and exposure technology; (**b**) DLP printing technology capable of seamless multi-material integration. Sources: [120,131].

In comparison, DLP technology achieves full-layer projection exposure based on digital micromirror devices (DMD), with all geometric features of each layer being cured and formed simultaneously. This surface exposure method makes its forming speed 5–10 times faster than SLA, and the speed is basically unaffected by the geometric complexity of the model. DLP 3D printing technology, based on light curing, utilizes ultraviolet light to initiate the polymerization of monomers and prepolymers, curing the resin layer by layer until the structure is formed. The core of DLP 3D printing technology lies in optical components, printing systems, and material systems, with materials generally being closely related to the printing system. As the core component of the DLP system, the micromirror size of the DMD has shrunk from the early 10 μm level to the current 5 μm level [114,115,116], meeting the basic requirements for the manufacturing of samples in the hundred-micrometer range [117,118,119]. Furthermore, the two-photon polymerization-enhanced DLP (TPP–DLP hybrid technology) can achieve a precision of the 100 nm level, expanding the limits of DLP technology [120,121]. In terms of light source technology, LED ultraviolet light sources with a wavelength of 405 nm have become the industry mainstream, with their advantages lying in high stability and long lifespan. It is noteworthy that multi-wavelength composite light source systems have emerged as the latest research direction. Dual-band or multi-band DLP printing equipment can simultaneously cure resin materials with different photosensitive groups, providing support for multi-material integrated printing [116,122,123]. As shown in Figure 5a, in terms of printing efficiency, modern DLP printing technology has achieved synergistic optimization of precision and efficiency through improvements in layering algorithms and exposure techniques [120,124,125]. Furthermore, by combining ionic gels with DLP technology, flexible ion–electronic devices and soft robots with pressure self-powered functions can be developed [6,96,126,127,128,129]. Combined with the design of the printing system, researchers have gradually achieved the printing of multiple materials such as hydrogels and resins. DLP 3D printing technology can seamlessly integrate different materials into a single printed structure (Figure 5b), is compatible with a wide range of materials from hydrogels to ceramics, and enables high-resolution, high-complexity, and high-speed 3D printing [97,130].

#### 2.1.3. Other 3D Printing Technologies

In addition to the commonly used MEX and VPP printing methods, there are sporadic reports on the preparation of flexible materials using PBF and BJT. Shuai et al. [132] employed a layer-by-layer preparation technique, utilizing TPU loaded with multi-walled carbon nanotubes (MWCNT) in combination with SLS, to fabricate porous scaffolds with shape memory functionality. These scaffolds exhibited excellent mechanical properties and biocompatibility. However, apart from the commonly used TPU material, these two processes are generally used to prepare materials such as alloys, and superelasticity and flexibility are achieved through structural design and other methods. For example, Zhen et al. prepared a novel shape memory alloy composition of Cu-18at%Al-10at%Mn-0.3at%Si, which exhibits excellent printability and adaptability in the laser powder bed fusion additive manufacturing process. The printed shape memory structures exhibit superelasticity and uniaxial and biaxial shape memory effects under different parameters [133]. In addition, BJT 3D printing technology is a promising additive manufacturing technology. It constructs three-dimensional objects by spraying liquid binder layer by layer to bond powder materials. The technical process mainly includes powder layering, binder spraying, drying and curing, and post-processing stages. The core lies in how to precisely deposit the binder droplets onto the powder bed and bond the powder through physical or chemical effects [134,135,136]. The main advantages of this technology include the following: (i) supporting complex designs, (ii) eliminating the need for support structures, and (iii) faster printing speed. It is compatible with a variety of materials, including polymers, metals, sand materials, and ceramics with different properties. The traditional BJT process is deeply tied to the post-processing (high-temperature sintering) steps for manufacturing rigid components (such as metals and ceramics), which fundamentally contradicts the characteristics of many flexible materials. However, BJT exhibits unique potential in the fabrication of rigid–flexible composite functional materials, especially in the field of flexible electronics [56,57,58]. For instance, BJT can be used to print metal powder green compacts, which, after sintering, form porous metal structures. These structures can then be infiltrated with flexible materials (such as polymers) to create metal–polymer composites, combining conductivity and flexibility [137]. By optimizing the powder and binder system, high-precision green parts are printed, and after sintering, a porous metal skeleton with interconnected pores is obtained. Subsequently, elastomers such as PDMS are infiltrated into the porous structure, thereby manufacturing a high-performance flexible strain sensor. This sensor exhibits good durability and sensitivity and can be used in fields such as robotic skin and health monitoring [138]. In recent years, BJT technology has made significant progress, but there are relatively few published works on using BJT technology to print flexible elastomers (such as soft materials similar to TPU). Further research is still needed to obtain the necessary basic data.

#### 2.1.4. Multi-Technology

Furthermore, researchers have significantly expanded the design and manufacturing boundaries in fields such as flexible electronics, biomedicine, and soft robots through strategies of collaborative manufacturing with multiple technologies and 3D printing, marking an important development direction for future advanced manufacturing. Collaborative manufacturing with multiple technologies and 3D printing is not simply a technical overlay, but rather a deep complementarity that leverages strengths and circumvents weaknesses [60,61,62,63,139,140,141,142,143]. Fuad et al. [59] prepared elastomeric PDMS with good biocompatibility by combining SLA technology with traditional techniques. Liu et al. [144] utilized the UV-followed curing method of UV-curable silicone rubber combined with DIW technology, ensuring the structural integrity of the fabricated silicone rubber parts without deformation. Yeong et al. [64] used FDM technology to print a macroscopic scaffold of biodegradable PCL with large pores and high porosity to provide mechanical support. Then, a layer of PCL nanofiber membrane was deposited on the surface and within the pores of the printed scaffold through electrospinning technology. The 3D printing contributes unparalleled shape complexity and customization capabilities, allowing the design and realization of complex three-dimensional structures that were previously “unmanufacturable”. Traditional or other technologies provide excellent resolution, material properties, or functional characteristics, ensuring the usability and high performance of the final devices.

### 2.2. Flexible Materials

In the field of additive manufacturing technology, flexible materials suitable for 3D printing have undergone significant phased evolution in recent years. In the initial stage, material research and development primarily focused on basic formability, aiming to achieve good print process adaptability. One example of this is ensuring the extrudability and interlayer bonding strength of TPE in FDM to meet the basic requirements of prototype manufacturing and simple model shape reproduction. Subsequently, the development focus gradually shifted towards performance modification, through means such as molecular structure design, filler compounding, and process parameter optimization, to specifically regulate the mechanical properties, thermal stability, fatigue resistance, and wear resistance of materials, making them suitable for industrial environments with more functional requirements, such as flexible fixtures and shock-absorbing components. Currently, this field is further expanding towards functionalization and intelligence, with a focus on developing multifunctional material systems that exhibit conductivity, biocompatibility, shape memory, self-healing capabilities, or the ability to respond to environmental stimuli such as temperature and pH. These advanced materials provide a crucial material foundation for emerging applications such as soft robots, wearable electronic devices, and biomedical devices, marking a new stage in flexible 3D printing that is moving from “making shapes” to “achieving functions”.

#### 2.2.1. Thermoplastic Flexible Materials

**Figure 6:**
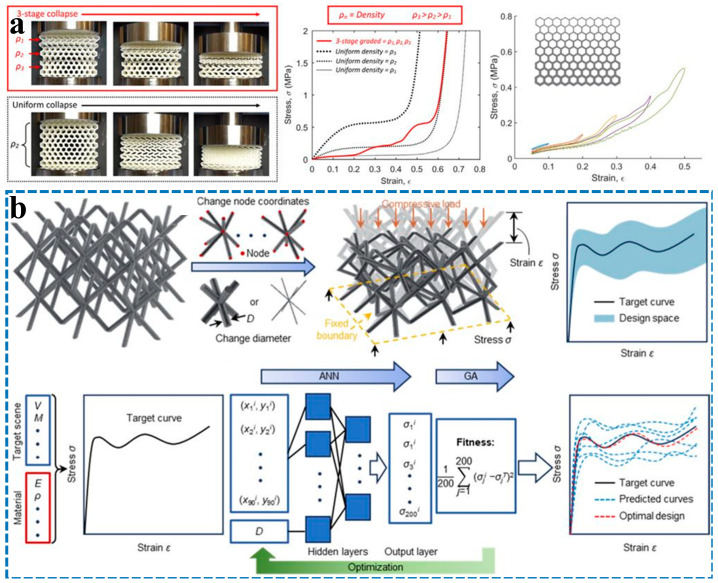
DLP printing technology optimized by utilizing artificial neural networks and genetic algorithms. (**a**) Flexural mechanical properties of optimized structures, (**b**) Schematic diagram of optimizing DLP printing process through genetic algorithm. Sources: [159].

The thermoplastic flexible materials for 3D printing primarily belong to the broad category of TPE. At processing temperatures, TPE exhibits the melt flow characteristics of thermoplastics and can be used on FDM and SLS 3D printers. At use temperatures, it displays elasticity and flexibility akin to vulcanized rubber [145,146,147,148,149,150]. Based on its chemical structure, the TPE commonly used for 3D printing is primarily TPU [151,152]. TPU is a block copolymer composed of hard and soft segments, whose hardness and properties can be precisely controlled by adjusting the ratio [150,152,153,154,155,156,157]. TPU possesses high impact absorption capacity and recyclability, making it suitable for applications requiring repeated bending or impact resistance. Researchers have fully utilized the resilience of TPU through methods such as origami design, foam filling, and gradient structure printing. For example, Simon et al. [158] explored the effects of various gradient forming methods on the energy absorption and damping properties of flexible TPU honeycomb structures. The developed 3D printing process can produce high-quality structures, with a maximum impact energy tolerance of 270 mJ/cm^3^ during cyclic loading densification, revealing the potential of TPU structural density gradient forming technology to provide excellent impact resistance protection under extreme environmental conditions. Furthermore, with the advancement of computer technology, by utilizing artificial neural networks and genetic algorithms (Figure 6), and combining the inverse design of energy absorption structures with the topological deformation of body-centered cubic (BCC) lattices, metamaterials with specific platform stress values (0.015 to 4.05 MPa) and specific energy absorption values (0.049 to 23.377 J/g) can be designed.

This approach precisely locates and optimizes parameters from an 181-dimensional space, fully leveraging the flexible and elastic properties of TPU materials [159]. However, thermoplastic TPU also has significant drawbacks: when printed using FDM technology, it poses higher difficulties due to the viscoelastic nature of the material, which can easily lead to feeding issues or nozzle blockages. When printed using SLS technology, the surface of the printed parts may appear slightly rough [159]. Moreover, compared to rigid materials such as PLA or ABS, it has higher costs [160,161].

In addition, styrenic elastomers (TPS, such as SBS) [162], thermoplastic copolyester elastomers (TPC) [146], and thermoplastic polyamide elastomers (TPA/PEBA) [163] can also be used as 3D printing filaments, providing excellent mechanical properties and temperature resistance. Among them, PEBA (Polyether Block Amide) is a high-performance polyamide elastomer, known for its high resilience (energy return rate), excellent fatigue resistance, and light weight. However, it is relatively expensive and is mainly used in high-end sports shoe midsoles, high-performance components, etc. [164,165,166].

#### 2.2.2. Light-Cured Flexible Resin Material

**Figure 7:**
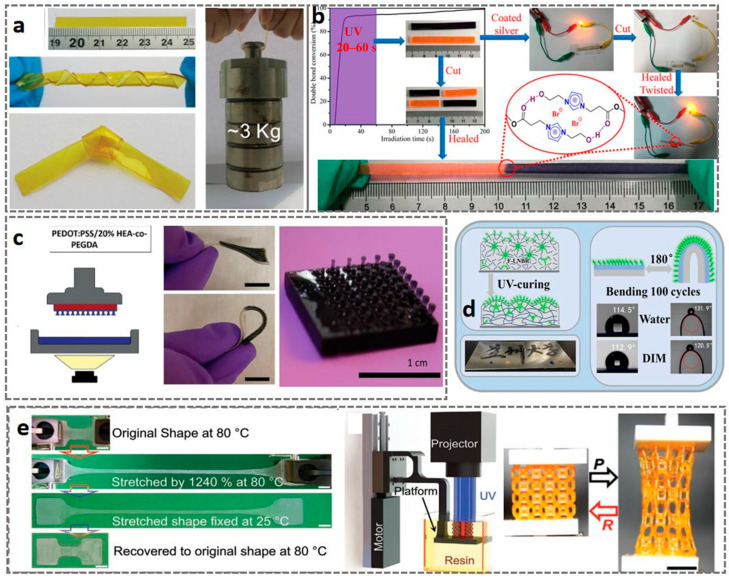
(**a**) A photopolymerizable ink based on acrylated polyethylene glycol (Acryl@PEG) for DLP 3D printing, exhibiting excellent elastic recovery; (**b**) a novel imidazole-containing photopolymerizable monomer for DLP 3D printing, serving as a self-healing polymer; (**c**) a hydrophilic silicone-based ink derived from amphiphilic silicone oligomers; (**d**) a transparent and flexible amphiphilic coating prepared through solvent-free coating and fluorinated liquid nitrile−butadiene rubber photopolymerization; (**e**) a shape memory functional variable polymer fabricated via DLP technology. Sources: [19,168,170,171,177].

The 3D printable light-cured flexible and elastic materials primarily belong to the photosensitive resin system based on acrylate or polyurethane acrylate. From the perspective of material types, these materials undergo crosslinking polymerization of active monomers/prepolymers in liquid resin through UV light, forming a three-dimensional elastic network structure, which is a thermosetting polymer. The materials commonly used are flexible photosensitive resin or rubber-like resin. Some high-performance systems may also contain components such as silicone-modified polyurethane acrylate to achieve a touch and performance closer to real silicone rubber [6,167]. Common UV-curable flexible resin materials include PUA, PEGDA, UV-PDMS, PC, Silicone Resin, NBR, epoxy resin, PI, etc. [168,169,170,171,172,173,174]. These widely used materials, when combined with UV-curable 3D printing technology, have seen significant demand in many fields. In terms of applications, thanks to the high-precision characteristics of UV-curing technology, these materials are particularly suitable for manufacturing flexible functional components with complex and fine structures. For example, in the medical field, it is used to print personalized soft tissue surgical guides, bionic anatomical models, and wearable rehabilitation devices [6,175]; in industrial design, it is used to produce flexible sealing rings, cushion pads, anti-slip grips, and soft robotic drive structures with high surface quality; in the consumer electronics field, it can also be used in prototype design to simulate soft-touch buttons and elastic shell components [167,176]. Furthermore, researchers have developed multifunctional flexible materials with hydrophilic flexible resin and self-healing capabilities through modification and synthesis processes [168,171,177] (as shown in Figure 7). Gong et al. designed and synthesized a novel imidazole-containing photocurable monomer. The prepared self-healing polymer, IB7-IM5, exhibited a tensile strength of 3.1 MPa, an elongation at break of 205%, and a healing efficiency of 93%, with a wide healing temperature range from room temperature to 120 °C (as shown in Figure 7b) [177].

However, commercial light-cured elastomeric inks used for 3D printing typically exhibit poor mechanical strength, poor resilience, and low elongation at break [6]. To address this, researchers have improved the mechanical properties and wear resistance of flexible materials through modifications and optimization of printing components. For example, as shown in Figure 7a, Ji et al. [170] developed a light-cured ink for digital light processing 3D printing using acryl-modified polyethylene glycol (Acryl@PEG). The resulting light-cured ink not only exhibited a high tensile strength of 14.1 MPa and an elongation of 245.0%, but also demonstrated excellent resilience (recovering to 90.85% after 30 min at 200% strain). Light-cured flexible elastomers typically exhibit low Shore A hardness (around 50–80A), high elongation at break (150–300%), and remarkable energy dissipation characteristics. Their tensile strength generally ranges from 1 to 15 MPa, which, although lower than that of high-performance thermoplastic elastomers, enables them to absorb energy during repeated deformation and provide excellent cushioning and sealing performance, sufficient to meet the needs of most non-load-bearing flexible scenarios [19,113,177,178,179].

#### 2.2.3. Hydrogel-Based Flexible Materials

**Figure 8:**
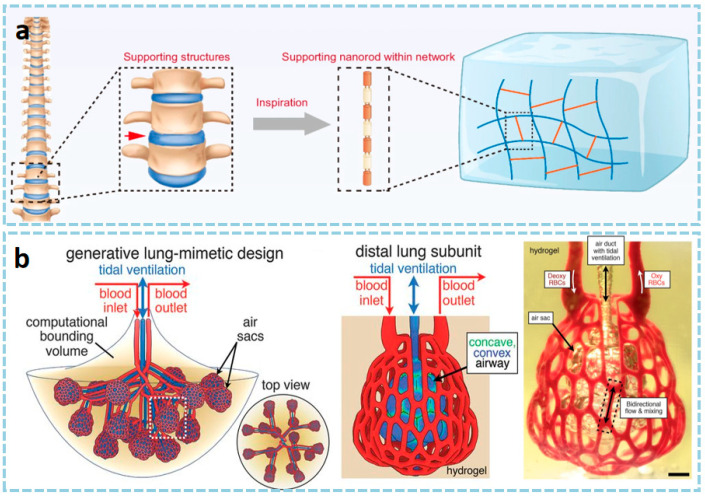
(**a**) Peptide-based rigid nanorod-enhanced gelatin methacrylate hydrogel; (**b**) monolithic transparent hydrogel containing highly efficient intravascular three-dimensional fluid mixer and functional dual valves (scale bar, 1 mm). Sources: [184,189].

The 3D printable hydrogels are a type of soft and wet material with a hydrophilic three-dimensional network structure. In recent years, they have attracted widespread research interest in the fields of biomedicine and flexible devices, and are one of the most feasible printing materials for manufacturing three-dimensional porous scaffolds [180]. From the perspective of the synthesis mechanism, hydrogels mainly include physical crosslinking, chemical crosslinking, and radiation crosslinking. From the perspective of material type, they can be mainly divided into natural polymers (such as gelatin, alginate, hyaluronic acid), synthetic polymers (such as polyethylene glycol (PEGDA)), and composite hydrogels (such as nanocellulose reinforcement systems) [181]. In recent years, with technological advancements, new types of hydrogels, such as supramolecular hydrogels and multifunctional responsive hydrogels, have gradually emerged [182,183]. Hydrogel materials can achieve high-precision molding through light curing (such as DLP and stereolithography SLA), MEX, or other technologies. In terms of materials used, chemically modified photocrosslinkable materials are particularly common. For example, methylacryloylated gelatin (GelMA) has become one of the standard materials in the field of tissue engineering due to its excellent biological activity and tunable physicochemical properties [184,185,186] (as shown in Figure 8a); poly(N-isopropylacrylamide) (PNIPAM) is widely used in tissue engineering, regenerative medicine, and 4D printing smart actuators due to its biocompatibility and temperature-sensitive properties [187,188,189].

In terms of applications, the core applications of 3D printed hydrogels are primarily focused on the biomedical field, with typical representatives including personalized tissue engineering scaffolds (such as cartilage, skin, and blood vessels), drug controlled-release systems, in vitro disease models, and recently emerging implantable flexible electronic sensors [189,190] (as shown in Figure 8b). In the field of soft robotics, hydrogels are also used to develop stimuli-responsive actuators. Regarding its mechanical properties, hydrogels typically exhibit a low elastic modulus (approximately 1–100 kPa), which can well simulate biological soft tissues; their elongation at break is mostly between 100–500%, indicating strong deformability but generally low toughness [191,192,193]. To this end, Bao et al. [194] composed an anti-tear hydrogel called DA@CNC, consisting of dopamine hydrochloride (DA)-modified N-isopropylacrylamide (NAM) and cellulose nanocrystals (CNC). This gel exhibits excellent mechanical properties, such as tear resistance, elasticity, and toughness. The introduction of DA@CNC not only endows the gel with substantial energy dissipation capacity through hydrogen bonding crosslinking, but also effectively inhibits crack propagation as a nanoscale reinforcement phase, thereby significantly enhancing the tear resistance of the gel. It is noteworthy that the mechanical behaviors (such as viscoelasticity, strength, and recoverability) of most hydrogels are strongly dependent on the crosslinking density, water content, and environmental stimuli (such as pH and temperature). This provides abundant possibilities for designing intelligent soft materials with tunable properties. For example, Wang et al. [195] developed an anti-swelling photocurable co-crystalline gel through the synergistic effect of hydrophobic/hydrophilic networks and metal coordination. The prepolymer mainly consists of 2,2,2-trifluoroethyl acrylate (TFEA), hydroxyethyl methacrylate (HEMA), acrylic acid (AA), zirconium oxychloride, and diethyl siloxane (DES) solvent. After photocuring treatment, the co-crystalline gel exhibits excellent mechanical properties, anti-swelling characteristics, environmental stability, conductivity, and sensitivity, making it suitable for smart wearable devices. Similarly, the increase in water content and tear resistance also poses special challenges to printing processes and structural stability [196].

#### 2.2.4. Flexible Composites

The 3D printable flexible composites are currently a research hotspot in the interdisciplinary field of soft materials and advanced manufacturing. The core of these materials lies in achieving synergy between function and structure through multi-material strategies or nanocomposite technologies. Especially in the fields of medicine and soft robotics, flexible composite materials are evolving towards functionality based on different application scenarios. In the medical field (such as implantable devices and surgical instruments [3,4], the main application requirements revolve around biocompatibility and safety. Flexible materials must remain stable in complex physiological environments, be non-toxic, and not trigger immune rejection. Therefore, materials with excellent biocompatibility, such as hydrogels and medical silicone [182,183], become the first choice. The technical challenges focus on precise manipulation and controllable degradation at the microscale, such as targeted drug delivery [189], and achieving mechanical compatibility with biological tissues, such as bionic vascular scaffolds [190], to avoid damage to surrounding tissues. In contrast, the demands in the field of soft robots emphasize macro functionality and environmental interaction capabilities. Materials need to possess rapid deformation recovery, high durability, and mechanical strength to withstand certain loads. Therefore, materials such as dielectric elastomers and shape memory polymers, which can generate large driving strains and forces, are highly favored [26,101].

From the perspective of material types, these materials are mainly divided into elastomer-based composites, hydrogel composites, and light-cured flexible resin composites.

- Elastomer-based composites

**Figure 9:**
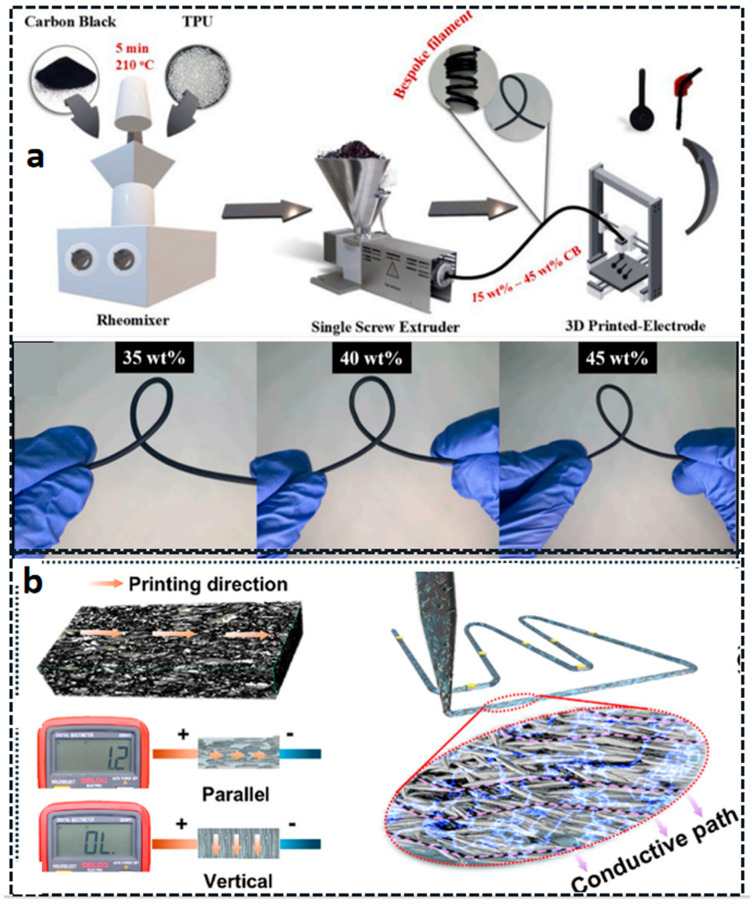
(**a**) TPU-based carbon black composite; (**b**) soft inductive coil composite. Sources: [198,200].

Elastomer-based composites are primarily formed by incorporating nano-systems and fiber systems into flexible elastic composites, such as TPU/nanoparticle systems and PLA/nanoparticles or fiber systems. They are mainly categorized into particle-filled elastomer composites, fiber-reinforced elastomer composites, and elastomer/thermoplastic blends. These composites are generally suitable for FDM and DIW 3D printing technologies. Particle-filled elastomer composites are the most traditional type, which enhance elastomers by incorporating micron- or nanometer-sized particles that effectively transfer loads through interfacial interactions and restrict the movement of polymer segments. The enhancement effect depends on the particle size, structure, surface chemistry of the filler, and the filler–matrix interaction [197,198] (Figure 9). Fiber-reinforced elastomeric composites primarily achieve significant anisotropy or isotropy enhancement for elastomers by incorporating chopped fibers or fiber fabrics as reinforcing skeletons, especially in terms of modulus and dimensional stability [199,200,201].

For instance, as shown in Figure 9b, Shan et al. [200] prepared high-stability carbon-fiber-reinforced liquid metal elastomer (CFLME) using an integrated approach: Ni plating on carbon fiber to enhance reactive wetting with liquid metal, followed by composite formation with elastomer and 3D printing for directional fiber alignment, yielding anisotropic CFLME. Such anisotropic architecture enables efficient conductive pathways along fiber axes, reducing the electrical percolation threshold to 25%, achieving a high electrical conductivity of 3.44 × 10^5^ S/m, and a thermal conductivity of 7.26 W/(m∙K). The fiber network securely locks liquid metal, enabling zero leakage under 400% strain, 1000-cycle stretching, or 833 kPa compression. Sisanth et al. [202] incorporated MWCNTs into silicone rubber matrices. Through rigorous experimental design and theoretical modeling, they revealed the complex relationship between MWCNT concentration, mechanical properties, and conductivity in the composite materials. As the MWCNT content increased, the improvement in tensile properties of the material demonstrated better dispersion effects and reinforcement effects, highlighting the potential for mechanical property optimization through regulation.

- Hydrogel composites

**Figure 10:**
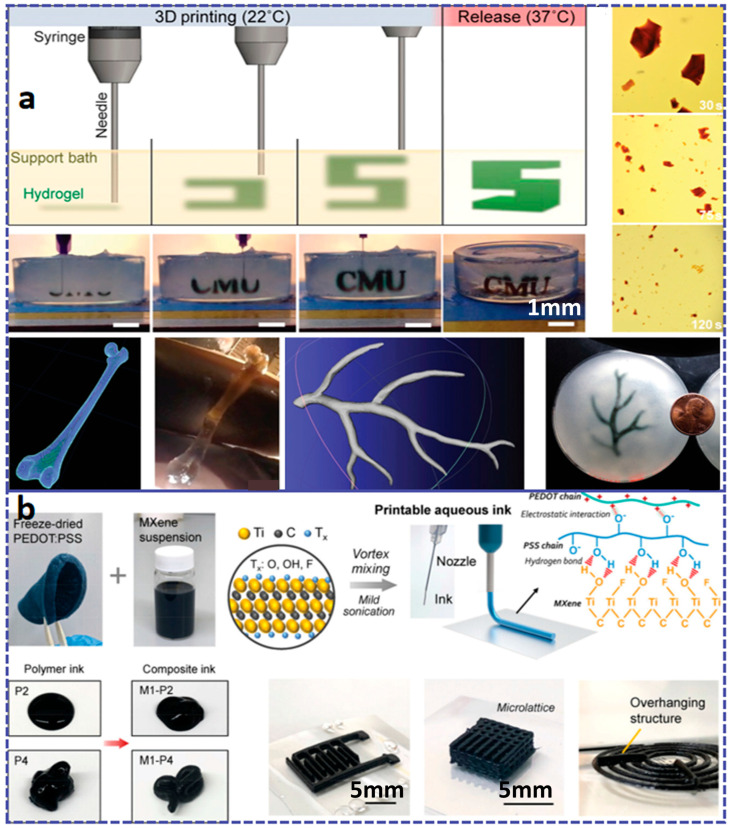
(**a**) Soft protein and polysaccharide hydrogel composites; (**b**) Ti_3_C_2_-MXene conductive hydrogel composites. Sources: [207,208].

Hydrogel composites primarily consist of inorganic nanoparticle/hydrogel composites, polymer network/hydrogel interpenetrating network composites, fiber/hydrogel composites, organic/inorganic hybrid elastomer nanocomposites, and biomacromolecule/hydrogel functional composite systems [14,203,204,205]. Hydrogel composites typically significantly enhance the mechanical properties, conductivity, antibacterial properties, or osteogenic ability of hydrogels by introducing inorganic nanoparticles. Nanoparticles, serving as crosslinking points or reinforcing fillers, physically or chemically interact with the hydrogel network. As shown in Shao et al. [206], 3D gel printing technology was successfully utilized to fabricate porous hydroxyapatite (HA) scaffolds for bone tissue engineering, providing channels for osteocyte adhesion, proliferation, and substance transport. As depicted in Figure 10a, Liu et al. [207] prepared a polymer network interpenetrating hydrogel with excellent biocompatibility and conductivity through freeze–thaw technology. This hydrogel can create 3D objects of arbitrary geometric shapes through extrusion printing. The obtained hydrogel exhibits a high conductivity of 1525.8 S m^−1^ and a water content of up to 96.6 wt%, demonstrating good flexibility, tensile strength, and fatigue resistance. As illustrated in Figure 10b, Thomas et al. [208] achieved 3D printing of hydrous materials such as alginate, collagen, and fibrin with an elastic modulus of <500 kPa through additive manufacturing techniques utilizing soft protein and polysaccharide hydrogels for complex three-dimensional biological structures.

- Light-cured flexible resin composites

Light-cured flexible resin composites primarily refer to flexible composites that incorporate functional fillers, such as carbon nanotubes (CNTs), graphene, liquid metal droplets, or magnetic particles, into flexible polymer matrices (such as TPU, SEBS, acrylate elastomeric resins) to impart electrical, magnetic, thermal, or mechanical reinforcement properties to the materials. Common flexible composites include elastomer/rubber-based light-cured composites, interpenetrating polymer network (IPN)-type light-cured flexible composites, functional filler/photosensitive resin composites, and degradable/photosensitive resin composites [209]. These composites are often fabricated using light-cured (DLP/SLA) 3D printing technology, and multi-scale structure manufacturing can also be achieved by combining FDM or DIW technology with UV technology.

Elastomer/rubber-based light-cured composites are based on resins with inherent flexibility, such as light-cured polyurethane acrylate (PUA), polyester acrylate, or hydrogenated nitrile rubber, as the matrix. Their mechanical properties or functions are further enhanced through the addition of composite fillers. The principle is mainly to utilize flexible molecular chains (such as polyether and polyester segments) as soft segments to provide elasticity, while fillers provide reinforcement or functionality. Li et al. [210] added acrylic urethane (UA) containing various monomers and monomer contents (CTFA, THFA, ODA, LA, IDA) into a container and obtained a mixed flexible light-cured resin through heating and stirring. Through DLP 3D printing, electrodes with octopus-like structures and pre-stretched wires were fabricated. Among them, UA, as an oligomer containing polyurethane chains, was combined with different light-cured monomers to obtain pre-light-cured flexible composites. Vincent et al. [211] published a bio-based acrylate photocurable resin formulation suitable for stereolithography 3D printing. The formulation was prepared by adding TPO initiator (0.40 *w*/*w*%) and BBOT absorber (0.16 *w*/*w*%) to a cylindrical polypropylene flask in a dark fume hood, and dissolving them in SA5102 acrylate monomer (19.9 *w*/*w*%) through vigorous stirring. Subsequently, SA5201 acrylate monomer (39.8 *w*/*w*%) and SA7101 acrylate oligomer (39.8 *w*/*w*%) were added to obtain a bio-based acrylate photosensitive polymer resin. This flexible composite resin not only enables high-precision printing of complex three-dimensional structures via SLA, but also exhibits a fracture stress value greater than 40 MPa.

Interpenetrating polymer network (ipn)-type light-cured flexible composites are formed by creating two or more interpenetrating polymer networks to synergistically enhance performance. The interpenetration of networks achieves complementary properties, such as one network providing strength and the other providing flexibility [212]. Meanwhile, with the development of dual-curing 3D printing systems, it has become possible to fabricate complex structures [213]. Obst et al. [214] analyzed the influence of light exposure on dual curing and the resulting material properties, and examined the network formation relationship between the secondary reaction of diaminopropylene diamine oligomers containing photocrosslinks and triamine components in the sequential crosslinking process. Forming interpenetrating polymer networks (IPNs) using epoxy–acrylate hybrid photopolymers and conducting 3D printing is a common approach. Redmann et al. [215] studied the two curing stages of dual-cured epoxy resin and conducted a kinetic analysis. They optimized the thermal curing process from 9 h to 3 h without affecting the thermomechanical properties, while maintaining the maximum conversion rate at a moderate value. This IPN structure effectively resolves the “strength–toughness” contradiction of single photocurable materials, achieving a combination of high strength and high toughness while reducing shrinkage stress [216].

Functional filler/photosensitive resin composites are prepared by directly adding functional micro- and nano-fillers to flexible photosensitive resins, resulting in flexible devices with functions such as flexible sensors, conductors, and actuators [217,218,219,220]. The resin matrix provides a flexible skeleton, while the functional fillers provide conductive, thermal, and magnetic response, and other properties. For example, using Fused Filament Fabrication (FFF) to print flexible thermoplastic polyurethane/multi-walled carbon nanotubes (TPU-MWCNT) composites, the nanocomposite with 3 wt% MWCNT exhibits repeatable and frequency-independent conductivity behavior, with maximum values reaching 0.10 and 0.32 S/cm, respectively, making it a new application in the fields of electronics and robotics [221]. In recent years, significant developments have been made in the field of electrically enhanced polymers and electromagnetic shielding interference in sensors. Researchers face many challenges in inducing conductivity in insulating polymers [152]. Various fillers such as CNTs, graphene, CCB, and polypyrrole (PPY) are used to enhance conductivity. These electrically modified TPUs have broad application areas, such as electronic watches, gas sensing, strain sensors, piezoresistive sensors, biomedical devices, robots, and wearable gloves. The potential uses of these sensors include soft robots, prosthetics, and wearable electronic products, as well as touch sensors, all of which require complex design, multi-directionality, embeddability, and customizability [219,220].

Degradable/photosensitive resin composites are a class of advanced functional materials that can be rapidly cured and molded through exposure to ultraviolet or visible light, while also exhibiting excellent flexibility, deformability, and the ability to gradually decompose into harmless small molecules under specific environmental conditions (such as aqueous solution, microbial action, or the internal environment) [222]. Their core definition encompasses two major elements: firstly, their curing process is photopolymerization, which offers advantages of high efficiency, good precision, and low energy consumption; secondly, their material sources or ultimate fate are environmentally friendly, either derived from renewable biomass resources (bio-based) or capable of natural degradation after use, aligning with the principles of circular economy and sustainable development. Focusing on biomedical and sustainable needs, these composites utilize biodegradable polyesters (such as polycaprolactone PCL) or derivatives of bio-based raw materials (such as soybean oil) as the matrix. By utilizing bio-based or degradable flexible oligomers, the materials achieve green and environmentally friendly properties [223,224]. Currently, this type of material system primarily utilizes biodegradable flexible polymers as photosensitive prepolymers, with the most typical representative being acrylate-terminated polycaprolactone (PCL-A). The soft segment of polycaprolactone provides excellent flexibility and degradability, while the terminal acrylate group endows it with photocurable properties. Additionally, oligomers derived from plant oils or bio-based monomers, such as soybean oil epoxy acrylate and itaconic acid-based polyester acrylate, have also become research hotspots due to their renewable sources [225,226,227,228]. In practical applications, these photosensitive resins are often compounded with various bioactive or degradable functional fillers, such as nano-hydroxyapatite (nHA) to enhance and impart osteoinductivity [229], cellulose nanofibers (CNF), or chitosan derivatives to improve mechanical properties and biocompatibility [230,231,232,233]. The application scenarios of these composite materials are highly concentrated in the biomedical field, especially demonstrating great potential in the manufacturing of personalized medical devices. They are widely used in light-curing 3D printing (such as stereolithography or digital light processing technology) to prepare various soft tissue engineering scaffolds (such as cartilage, blood vessels, and skin substitutes), customizable bioresorbable flexible implants (such as soft tissue fixation anchors or staplers), and drug controlled-release carriers [234,235]. Their value lies in the ability to quickly print personalized implant devices based on patients’ medical imaging data. These devices match native soft tissues in mechanical properties (modulus in the megapascal range), promote cell growth in the microstructure, and ultimately do not require secondary surgery for removal. This achieves full-process biomedical innovation from “structure and manufacturing” to “function and outcome” [32,236].

Currently, the applications of flexible composite materials have far exceeded the scope of traditional structural components, extensively covering emerging frontier fields, including wearable health monitoring electronic devices, soft robot perception and actuation systems, bionic artificial muscles, and tissue engineering scaffolds. In terms of mechanical properties, composite strategies have significantly enhanced the performance limits of single flexible materials: for example, nano-fillers can greatly increase the modulus (from several MPa to tens of MPa) and tear strength of elastomers; directional structural design combined with stretchable conductor embedding can achieve stable electrical resistance performance within a strain range of 20–500% [237,238,239]. However, the mechanical behavior of these materials also exhibits significant nonlinearity, viscoelasticity, and anisotropy, and their performance is highly dependent on interfacial bonding, filler distribution, and printing process parameters. These are key issues that require collaborative optimization in design and application. Related challenges and regulation strategies have been extensively explored in multiple studies in recent years [150,240].

Finally, we have summarized the main information about flexible materials, as detailed in Table 2.

## 3. Challenges Towards 3D Printed Flexible Materials

With the advancement of materials science and innovation in printing technology, multi-material printing technology and the improvement of printing accuracy and speed will further promote the expansion of flexible material applications. The medical, electronics, and consumer goods sectors will continue to deepen their applications, while emerging fields such as aerospace and automotive manufacturing will also become exploration directions. However, 3D printed flexible materials still face many technical challenges, including printing accuracy issues, material fluidity and deformation control, complexity of support structure design, and optimization of post-processing techniques. Solving these problems requires the development of high-precision equipment, the fine adjustment of printing parameters, and innovative support structure design [241,242,243].

The core technical challenges faced by 3D printing of flexible materials are primarily concentrated in three key areas: precision control, rheological behavior regulation, and post-processing optimization. In terms of printing precision, the high elasticity and low modulus characteristics of flexible materials lead to dimensional deviations during the molding process. The rheological properties of the material have a significant impact on the molding quality, and the challenges in the post-processing step are particularly prominent. Traditional support removal methods often damage the flexible substrate and result in a high residual rate, while secondary curing of light-cured materials often leads to an additional shrinkage of 3–5% and changes in mechanical properties. Currently, researchers have addressed these issues by developing low-shrinkage photosensitive resins, innovative support materials, and precise post-curing systems. However, achieving high-precision, high-performance flexible device manufacturing still requires breaking through the technical bottleneck of material–process–equipment collaborative optimization [244,245,246].

### 3.1. Challenges Towards Material Extrusion 3D Printed Flexible Materials

**Figure 11:**
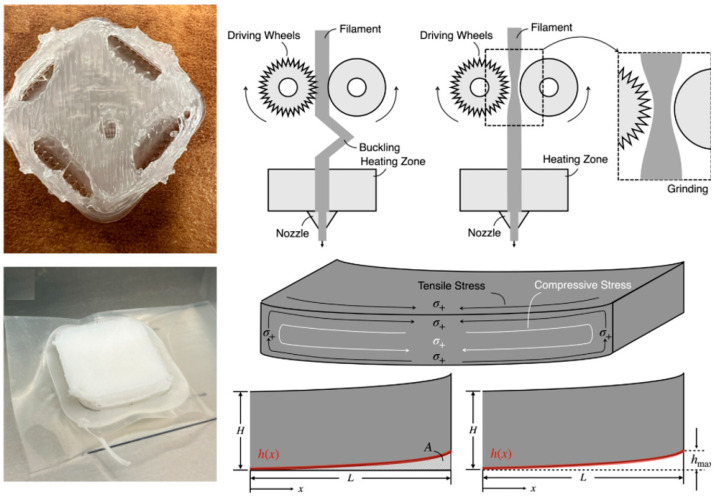
Potential defects in FDM printing. Sources: [249].

The defects of Fused Deposition Modeling (FDM) technology are primarily focused on accuracy, strength, and process stability, but they can be significantly improved through material science, algorithm optimization, and hardware innovation. Future research directions include intelligent process closed-loop control and the development of high-performance flexible materials. However, this technology has obvious process defects:

(1) Striped texture often appears on the surface of Fused Deposition Modeling (FDM) printed parts. Insufficient interlayer bonding can lead to rough surfaces, while uneven cooling shrinkage can cause sample deformation and warping, significantly influenced by nozzle extrusion control and temperature fluctuations [247,248,249], as shown in Figure 11. (2) Weak interlayer bonding can result in the insufficient mechanical strength of the sample, with Z-axis strength typically 30% to 50% lower than that of the X–Y plane [250]. (3) Complex structures rely on supports and pose post-processing challenges. Some overhanging structures require support materials, but removing the support can easily damage the model surface. (4) Material limitations exist, as different materials have different melting temperatures and cooling rates [251,252]. Improper temperature settings (e.g., PLA requires 190–220 °C) or doping with functional phases can easily cause nozzle blockage [253] (Figure 11). The key technical aspect of FDM technology is to maintain the temperature of the raw material ejected from the nozzle in a molten state slightly above its freezing point, typically within a range of 5 to 10 °C above the freezing point. If the temperature is too high, it can lead to delayed solidification of the material, resulting in issues such as model deformation and low surface precision. However, if the temperature is too low or unstable, it can easily cause nozzle blockage, leading to printing failures [86]. Additionally, the interlayer bonding strength of flexible materials prepared by FDM is only 30–50% of that of the injection molded parts, resulting in significant anisotropy (the tensile strength in the Z direction is 60% lower than that in the X–Y direction). The contradiction between printing speed and precision is prominent, with surface roughness Ra reaching 20–50 μm during high-speed printing (>50 mm/s). Furthermore, it is difficult to manufacture fine structures with feature sizes < 0.5 mm, all of which limit the development of FDM technology [254,255].

To address the shortcomings of Fused Deposition Modeling (FDM), researchers have embarked on improving FDM technology by integrating multifunctional printheads with advanced materials research and development efforts [256]. There are mainly four aspects: (1) Process parameter optimization and intelligent control. By maintaining a constant temperature of ±0.5 °C in the print chamber, thermal deformation is reduced. Meanwhile, machine learning algorithms and simulation modeling are combined to optimize the print extrusion path and enhance surface precision [257,258,259]. (2) Material modification. Researchers enhance the interlayer bonding strength by incorporating carbon fibers or nanoparticles (such as graphene) into flexible materials [76]. (3) Hardware improvement. For example, vibration isolation technology is employed to reduce the impact of mechanical vibration on interlayer adhesion. (4) Multi-nozzle system: It supports the simultaneous printing of water-soluble support materials and functional materials, reducing the difficulty of post-processing [247,260,261,262,263,264].

**Figure 12:**
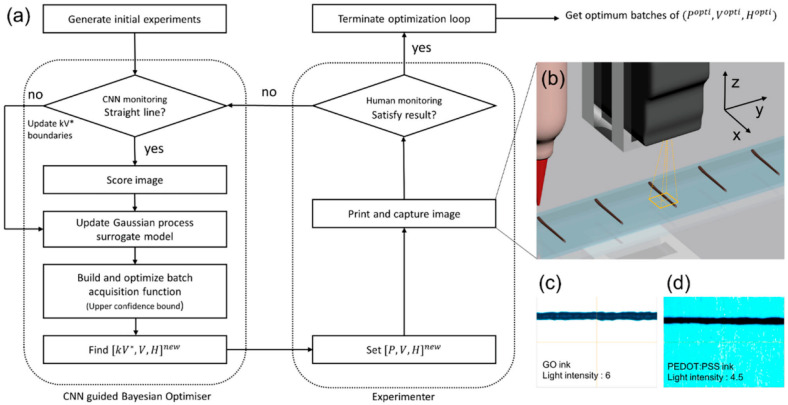
Introducing convolutional neural networks to optimize the 3D printing process. (**a**) CNN guided Bayesian optimiser and experimenter’s iteration process. (**b**) Capturing line image from the camera. Captured line images of (**c**) GO and (**d**) PEDOT:PSS inks. Sources: [271].

The core defect of DIW technology mainly lies in its stringent requirements for ink viscosity, which must exhibit both shear thinning and rapid curing capabilities. Too low a viscosity can lead to structural collapse, while too high a viscosity can make it difficult to extrude, affecting the accuracy of the final shape. Moreover, delamination is particularly prone to occur during the printing of flexible materials. Its main defects are manifested as follows: (1) limited rheological properties of the ink; (2) weak interlayer bonding and structural deformation, where the ink is accumulated layer by layer during printing, and the incomplete curing of the underlying ink may lead to the collapse of overhanging structures, especially when overhanging at large angles (>30°) [265]; (3) poor compatibility with multiple materials, as DIW typically requires optimizing parameters for a single ink, making it difficult to achieve interfacial bonding in multi-material printing, such as unstable electrical properties in conductive–insulative composite materials [90,266,267]. However, researchers have made improvements to address these deficiencies: (1) Optimization of ink formulation. For instance, multi-level particles are used for matching printing when configuring the ink. (2) The use of prepolymer diluent, adding multi-component resin to the light-cured ceramic ink to reduce the curing shrinkage rate [268]. (3) Process innovation. By designing a distance-controlled direct writing (DC-DIW) device, the interlayer distance is dynamically adjusted to achieve 30° overhanging structure printing of titanium alloy, reducing the need for support. (4) Multimodal printing: Combining DIW and light-curing technology to simultaneously form complex geometries and functional gradient materials. (5) Intelligent control. Real-time rheological monitoring: By adjusting the extrusion pressure (such as 0.1 MPa ± 0.02 MPa) and speed (10 mm/s) through sensor feedback, the uniformity of the ink is ensured [269,270]. For instance, as shown in Figure 12, a Bayesian optimization framework guided by a Convolutional Neural Network (CNN) is introduced to maximize the surface-to-volume ratio of 3D printed lattice supercapacitors. The linear classification CNN model guides the optimizer’s search space to the linear printing region, thereby minimizing optimization time and cost. The results are compared with parameters following the traditional DIW 3D printing method. The irregularity decreased by 61.8% and 18.9%, respectively, and the average width decreased by 39.0% and 28.6%, respectively [271]. It is worth noting that the current optimization of DIW defects mainly focuses on printing fields such as ceramics and metals, with less research on the optimization of flexible materials.

### 3.2. Challenges Towards Vat Polymerization 3D Printed Flexible Materials

VPP technology imposes strict requirements on the rheological properties and curing characteristics of photopolymer resins. Certain functional materials (e.g., high-filling functional phase particle slurry) face compatibility challenges due to issues such as light scattering or sedimentation. However, significant improvements can be achieved through nanocomposites, high-precision processes (e.g., two-photon printing), and intelligent control systems (e.g., IsT-VPP).

**Figure 13:**
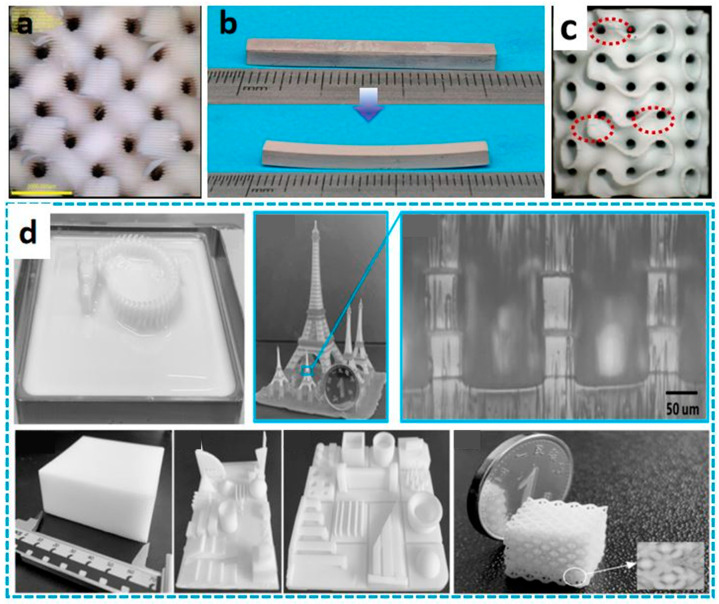
Typical defects in DLP printing. (**a**) Steps between cured layers; (**b**) residual stress; (**c**) cracking between cured layers; (**d**) sample size limitations. Sources: [282,283].

As shown in Figure 13, the core defects of VPP technology primarily manifest as follows: (1) Staircase effect and surface roughness. The layer-by-layer curing characteristic leads to staircase defects on curved or inclined surfaces, which significantly affects the surface smoothness of high-precision components such as optical lenses [272,273,274]. (2) Residual stress and deformation. During the photopolymerization process, monomer shrinkage (with a shrinkage rate of up to 5–10%) generates internal stress, leading to warping or cracking in thin-walled structures [275,276]. (3) Multi-material printing is challenging. The incomplete material switching mechanism, combined with variations in viscosity and curing rates among different resins, easily leads to weak interfacial adhesion or contamination [277]. Although VPP 3D printing technology has made significant progress, several critical challenges remain to be addressed. The most pressing issue is the interfacial adhesion problem in multi-material printing. Most multi-material switching processes require direct contact with solid wipers or fluid flow on printed components [278], which causes issues such as small sample sizes, limited material options, and severe material contamination in DLP-based multi-material 3D printing [279,280]. The bonding strength between different material regions remains 30–40% lower than that of homogeneous materials, which may become the source of failure under long-term cyclic loading. The intensity gradient distribution at material interfaces causes discontinuous crosslinking density, representing the fundamental reason for interface weakening that requires the development of novel photoinitiator systems and interface coupling agents [281,282,283]. (4) Printing efficiency contradicts large-scale structure fabrication. Although VPP has developed DLP planar exposure technology that achieves higher efficiency than point-scanning SLA, challenges persist during large-sized part printing. When forming dimensions exceed 200 mm, intensity uniformity differences between the edges and the center reach 15–20%, leading to inconsistent curing depths [279,280,284]. Some industrial-grade equipment employs multi-projector stitching technology to alleviate this issue, but this increases system complexity and costs. Future approaches may need to integrate adaptive optics and real-time monitoring technologies to overcome dimensional limitations while maintaining precision. (5) Environmental Impact of Post-Processing Processes. Traditional photopolymer resins in 3D printing post-processing typically require organic solvents for cleaning, generating hundreds of tons of toxic chemical-containing waste liquid annually during this process, which poses significant threats to ecological environments and human health [285].

Fortunately, researchers have conducted extensive studies on these issues, with the VPP 3D printing material system being a key breakthrough over the past decade. Currently, major optimization studies include the following:

(i) Material Innovation Optimization

**Figure 14:**
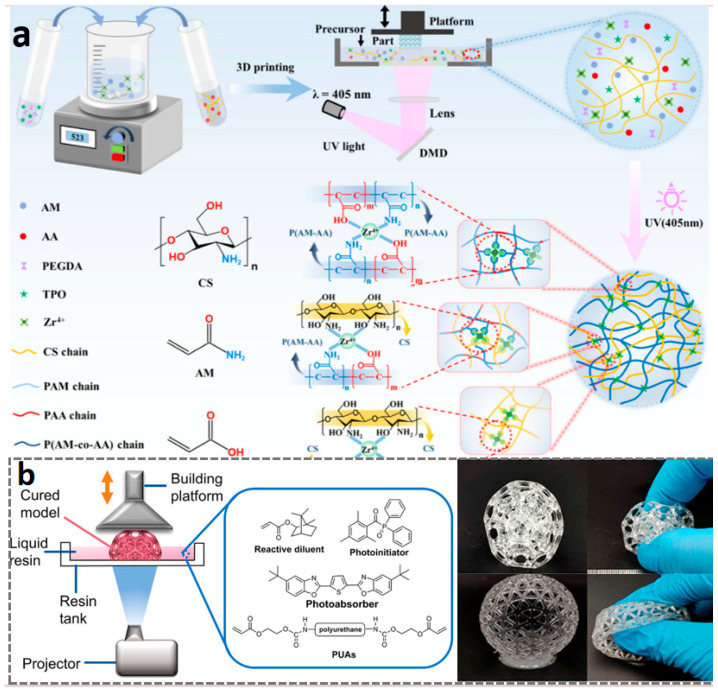
(**a**) Dual crosslinked hydrogel prepared by reductive polymerization 3D printing technology; (**b**) polyurethane elastomer with low viscosity and good mechanical properties. Sources: [287,288].

Traditionally constrained by acrylate and epoxy resin systems, the material family has now evolved to include ceramic slurries, hydrogels, and conductive polymers. The development of 3D printed polyurethane (PU) composites reinforced with zinc oxide (ZnO) nanoparticles, stabilized through surface functionalization using the silane coupling agent 3-(trimethoxysilyl)propyl methacrylate (TMSPM), demonstrates this advancement. The incorporation of TMSPM-modified ZnO nanoparticles significantly improves the uniformity of nanoparticle dispersion and the interfacial compatibility between inorganic fillers and polymer matrices. Compared to controls, ZnO-reinforced scaffolds retained over 75% of their mechanical properties while maintaining up to 53% compressive strength after 150 h of UV and thermal aging, opening new pathways for DLP 3D printed flexible sensors [281]. In addition, significant progress has been made in the field of biomedicine regarding DLP-compatible bioinks. For instance, Zhou et al. [286] developed a method to rapidly localize clusters of highly viable human skin fibroblasts (HSF) and human umbilical vein endothelial cells (HUVEC) using DLP-based 3D printing technology to form fibrovascular structures (FLS). These FLS structures promote skin regeneration and efficient neovascularization by mimicking the physiological architecture of natural skin. Their robust mechanical and bio-adhesive properties also enable facile handling and implantation at wound sites. Notably, as shown in Figure 14, Zhang et al. [287] realized a 3D printable hydrogel with excellent mechanical properties and conductivity through a simple one-step preparation strategy. The hydrogel can be cured based on a hybrid double network mechanism involving in situ chemical and physical double crosslinking. The hydrogel has good mechanical properties (680% tensile property, 15.1 MJ/m^3^ toughness, and 7.30 MPa tensile strength), fast printing speed (0.7~3s/100 μm), high transparency (91%), and good ionic conductivity (0.75 S/m). Peng et al. [288] successfully synthesized three polyurethane acrylate oligomers and prepared a low-viscosity UV-curable resin for digital light processing three-dimensional (3D) printers, without the need for customized equipment. The results showed that the resin exhibited excellent mechanical properties and shape recoverability, with a tensile strength and elongation at break of 15.7 MPa and 414.3%, respectively. Furthermore, it was capable of withstanding 100 compression cycles at 80% strain without fracturing.

(ii) Multi-Material System Development

The breakthrough in multi-material VPP printing technology is particularly notable. For example, full-arch denture manufacturing can now be completed in a single process, enabling simultaneous processing of regions with hardness variations ranging from Shore A 30 to 90, compressing the traditional multi-day manufacturing process into 24 h. This multi-material integration technology not only applies to dental applications but also provides manufacturing solutions for fields requiring mechanical gradient structures, such as flexible robots and wearable devices [277]. Tsai et al. [289] developed a novel resin formulation method that creates 3D printed conductive structures using digital light processing (DLP) 3D printing technology. Their approach employs AgCu as a conductive filler mixed with photocurable acrylic resin, while adding carbon nanotubes (CNTs) as a thickener to establish a support network that prevents metal filler settling. This multi-material resin can print 3D metal circuit structures with conductivity as high as 1000 S/cm without sintering, achieving multifunctional integration of ultra-devices.

**Figure 15:**
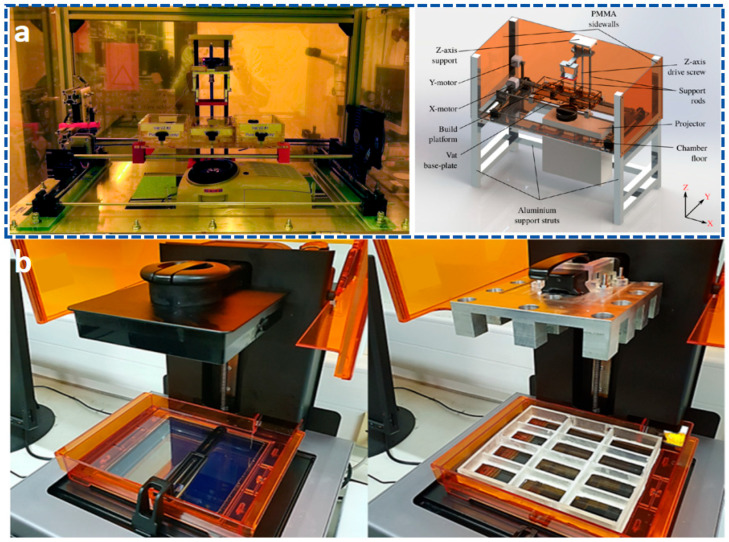
(**a**) Multi-material SL printing device; (**b**) stereoscopic lithography technology capable of simultaneously printing up to 12 different formulations. Sources: [290,291].

In addition, the capability of multi-material 3D printing can significantly enhance the performance and functionality of printed 3D objects, even achieving functionalities and properties that cannot be attained by single-material printing structures. Multi-material 3D printing systems consist of two main components: a manufacturing center and a control center. Researchers primarily achieve printing by manipulating the switching of different resin tanks, considering multiple aspects including material combinations, potential for new material development, multi-material printing, geometric resolution, machine investment, and consumables, thereby developing multi-material photopolymerization printing systems [290] (Figure 15a). For instance, as show in Figure 15b, Curti et al. [291] developed a novel SLA device specifically designed for rapid and efficient screening of drug photopolymer formulations. By designing and manufacturing a new resin tank and assembly platform, the commercial SLA equipment was improved. This platform can simultaneously 3D print up to 12 different formulations, reducing the required sample resin volume by 20 times. Using this improved SLA device, it has been confirmed that its time efficiency has been increased by 91.66% and 94.99% to achieve high-quality, high-printability output. This proves that such improvements provide a robust and reliable tool for optimizing the throughput and efficiency of liquid barrel photopolymerization technology in the formulation development process, thereby supporting future clinical applications. However, several issues remain to be resolved, such as laser blocking and material contamination, which limit the design flexibility of Multi-Material structures.

(iii) Process Optimization and Intelligent Post-Processing

**Figure 16:**
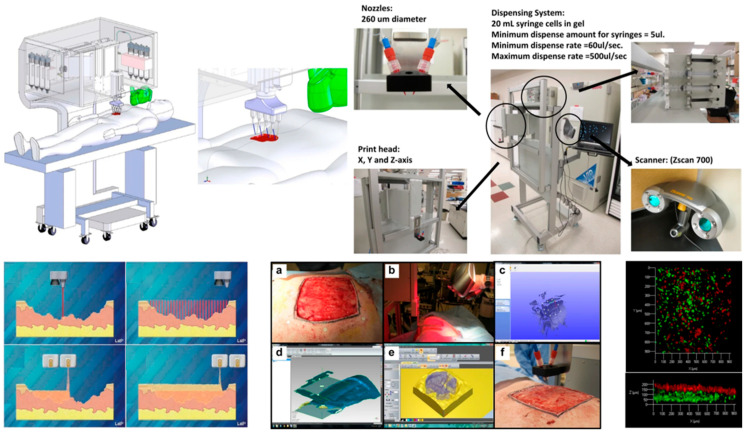
A mobile skin bio-intelligent printing system for rapid on-site treatment of large-area wounds. (**a**) Mark the position of the skin gap before scanning; (**b**) use the scanner to scan; (**c**) generate an STL file from the scanned information; (**d**) generate information such as the spray point path; output the information as code to the printer interface, and generate the nozzle path required for printing (**e**,**f**). Sources: [295].

The process optimization of VPP printing technology focuses on exposure parameters, material formulations, and support structure design to enhance printing accuracy, efficiency, and mechanical performance. Intelligent post-processing integrates machine vision and machine learning algorithms to automatically perform cleaning, secondary curing, and surface treatment, while real-time monitoring and parameter feedback regulation significantly reduce manual intervention and ensure batch consistency [292,293,294]. As shown in Figure 16, Albanna et al. [295] designed and validated a mobile skin bioprinting system capable of rapid on-site management of large-area wounds. Integrated imaging technology enables precise delivery of autologous or allogeneic dermal fibroblasts and epidermal keratinocytes to injured regions, replicating the layered skin structure. Wounds printed with layered autologous dermal fibroblasts and epidermal keratinocytes in hydrogel carriers demonstrated rapid closure, reduced contraction, and accelerated re-epithelialization. The deep integration of process optimization and intelligent post-processing is driving VPP technology toward full-process digitalization and intelligent development, laying the foundation for its large-scale application in medical, aerospace, and other high-end fields.

### 3.3. Challenges Towards Other 3D Printed Flexible Materials

In addition, 3D printing technologies such as powder bed fusion (PBF) and BJT are commonly used for printing flexible materials. Among these, the root cause of defects in PBF technology lies in the coupled material–process–thermodynamic issues, with defects primarily manifesting as the following: (i) Porosity defects (voids, lack of fusion). Excessive laser energy input (e.g., keyhole effect) or insufficient energy (incomplete melting of powder) creates voids, reducing the part density and mechanical performance. Voids and lack of fusion arise mainly when laser scan spacing or layer thickness is excessive, resulting in insufficient bonding between powder layers. (ii) Residual stress and deformation. Rapid solidification during processing accumulates thermal stress, causing warping or cracking in components, particularly pronounced in large-scale thin-walled structures. (iii) Spatter and globbing phenomena. Excessive laser power or unstable protective gas flow causes molten pool spatter that contaminates the powder bed, forming globbing defects. As analyzed by Leander et al. [296], extensive studies on material properties in thermoplastic TPU laser sintering include powder flowability, melt rheology, and shrinkage hardening behavior. Their research revealed that spatter during sintering can block laser beams, increasing porosity in subsequent layers. (iv) Powder contamination and compositional inhomogeneity. Dust contamination is an inevitable issue in laser sintering. In multi-material composite fabrication, uneven mixing often occurs.

However, researchers have employed intelligent regulation (e.g., online monitoring), material innovation (e.g., nanoparticles), and multiphysics simulation approaches to significantly improve defects. The main technical methods include the following: (i) Process parameter optimization and intelligent control. For instance, precise energy density regulation through experiments and simulations determines optimal laser power and scanning speed to reduce keyhole and lack-of-fusion defects, or multiphysics simulation methods guide the prediction of molten pool dynamics and optimization of scanning paths. (ii) Material modification. Material modification serves as a crucial approach to suppress particle splashing and ensure stable structural formation. (iii) Online monitoring and closed-loop control. AI image analysis identifies unfilled powder areas and enables real-time defect detection. (iv) Post-processing technological innovation. For example, staged temperature control reduces residual stress, enhancing sample precision and mechanical properties.

BJT technology offers advantages such as high printing efficiency and relatively low cost, as its printing process does not require high-power energy sources (such as lasers or electron beams), enabling rapid layering and jetting. It eliminates the need for support structures during printing because the loosely packed powder layer provides natural support, allowing the fabrication of highly complex geometries. Additionally, BJT materials can achieve multi-material printing through powder replacement or multi-head spraying of different binders. It demonstrates significant advantages in manufacturing large-scale parts. However, BJT technology has notable limitations: (i) Low green body strength, which heavily depends on binder adhesion. (ii) Difficult densification, resulting in challenging production of fully dense parts with inherent porosity. (iii) Surface roughness inherent to the BJT process. (iv) Poor multi-material compatibility. To address these drawbacks, researchers have developed various optimization techniques to enhance printing precision. Key approaches include the following: (i) Binder formulation optimization through nanocomposite binders and UV-curable systems to improve interparticle adhesion, combined with UV curing to reduce shrinkage effects. (ii) Process parameter optimization using multi-head collaborative spraying to control binder jetting dimensions and surface accuracy, along with intelligent optimization algorithms guided by software to refine structural fabrication. (iii) Material system expansion. By using BJT to apply graphene oxide (GO) as a binder for polyvinyl alcohol (PVOH) powder printing, conductive and highly flexible GO/PVOH composite materials are obtained [297]. The defects of BJT technology in flexible material fabrication lie in mechanical properties, accuracy, and post-processing deformation, but can be significantly improved through nanomodified binders, multi-head high-precision control, and intelligent simulation.

The core challenges in 3D printing of flexible materials primarily revolve around three critical aspects: precision control, rheological behavior regulation, and post-processing optimization. Regarding printing precision, the high elasticity and low modulus characteristics of flexible materials lead to dimensional deviations during shaping. For instance, photopolymerized elastomers exhibit microstructural deformation due to curing shrinkage rates, while TPU materials in FDM processes experience interlayer misalignment of 50–100 μm caused by rebound effects [72]. The rheological properties of materials significantly impact shaping quality. Although the shear-thinning effect of hydrogels facilitates extrusion, it often results in structural collapse, particularly with high-aspect-ratio microstructures that show low successful fabrication rates. High-viscosity elastic resin (>5000 cP) faces switching inefficiency and cross-contamination issues during multi-material printing. Post-processing challenges are particularly pronounced: conventional support removal methods damage flexible substrates with high residue rates, secondary curing of photopolymer materials commonly induces additional shrinkage and mechanical property changes, and functionalized flexible materials (e.g., embedded electronics) experience performance degradation when post-processing temperatures exceed 80 °C [251,252].

In terms of printing accuracy, the inherent low modulus and high ductility of flexible materials make dimensional control during the forming process particularly challenging. The internal stresses generated during curing shrinkage of light-curable flexible resins can cause significant deformation in micrometer-scale structures (e.g., bio-inspired micropillar arrays), with typical deviations reaching 10–15% of the designed dimensions [293]. Meanwhile, the rebound effect of elastic materials like TPU in fused deposition modeling (FDM) processes leads to serpentine distortion in extrusion paths, particularly when printing suspended structures, where interlayer misalignment can reach 50–100 μm [251]. Current solutions to these precision issues primarily focus on two directions: intelligent compensation algorithms and development of novel material systems. Examples include using machine-learning-based deformation prediction models for pre-compensation of light-curing paths, or creating photocurable elastomers with shape memory characteristics to suppress curing shrinkage. Some advanced processes have already achieved forming accuracy of complex flexible structures within ±20 μm [97].

The precise control of material rheological behavior represents another core challenge in flexible material 3D printing, as these materials typically exhibit significant non-Newtonian fluid characteristics and time-dependent properties. While the shear-thinning effect observed in biocompatible materials like hydrogels during extrusion facilitates passage through narrow nozzles, their slow structure recovery characteristics cause printed three-dimensional network structures to collapse under self-weight, particularly when characteristic dimensions fall below 100 μm. For instance, sodium alginate/GelMA composite hydrogels experience over 90% structural collapse rates due to delayed modulus recovery during high-aspect-ratio structure printing [184,185]. In photopolymerizable flexible materials, resin viscoelasticity directly impacts liquid–air interface separation and coating quality, with high-viscosity elastic resins commonly exhibiting low switching efficiency and material mixing contamination in centrifugal multi-material printing systems. Each material switching requires 30–60 s of cleaning time, severely constraining printing efficiency [279,287]. In the field of material development, current research focuses on two directions: biomimetic design and nanocomposites. Dynamic covalent bonds or nanocellulose as reinforcing phases are introduced to regulate rheological properties [277,288]. In terms of process innovation, emerging acoustic-field-assisted and magnetic-field-directed technologies provide new approaches for rheological control. For instance, ultrasonic vibrations can transiently reduce the apparent viscosity of bio-ink, while magnetic fields can induce the directional alignment of anisotropic fillers during extrusion. These methods significantly improve manufacturing quality without altering the material formulation [298,299,300,301].

The impact of post-processing on the final performance of flexible materials is often underestimated but crucial. The removal of support structures represents the first challenge, as traditional water-soluble or thermally meltable support materials may cause mechanical damage to the flexible substrate during removal, particularly affecting devices with microchannel or cavity structures, which can severely compromise device functionality. Post-curing processes face more complex challenges, as the secondary curing of photo-cured elastomers not only induces additional volumetric shrinkage but also alters material mechanical properties. Current trends in post-processing technology development include the following: intelligent process control, such as precision post-curing systems with real-time infrared monitoring that dynamically adjusts light intensity and temperature based on material conditions [302]; and green solutions, such as recyclable support materials based on ionic liquids. Notably, synergistic optimization between post-processing techniques and printing parameters is becoming increasingly important. By considering post-processing effects during design phase-optimizing model orientation to reduce support requirements, or adjusting fill patterns to balance curing uniformity, the consistency of final product performance can be significantly enhanced.

### 3.4. Challenges Towards Economic Cost

The 3D printing of flexible materials also faces numerous challenges on the economic front, particularly in terms of implementation costs and technological scalability.

The price range for 3D printing equipment for flexible materials is wide: from desktop-level FDM printers (for TPU/TPE) priced at a few thousand dollars to industrial-grade multi-material SLS printers costing hundreds of thousands or even millions of dollars. Furthermore, due to the high printing difficulty of flexible materials, they require higher stability and precision from the printing hardware, which directly drives up costs. For instance, in the feeding system, flexible filaments are prone to bending and getting stuck in FDM printers, necessitating the use of printers equipped with direct-drive extruders, which are more expensive than standard setups. For nozzles, printing flexible materials, especially those containing fillers such as carbon fibers, exacerbates nozzle wear, requiring the use of hardened steel or gemstone nozzles, which is an ongoing consumable cost. Additionally, there is the important temperature control system: industrial-grade equipment requires more precise heating bed and chamber temperature control to ensure interlayer adhesion and reduce warping.

Meanwhile, compared to ordinary resin materials, flexible materials have a higher unit price: the research and development (R&D) and production costs of flexible polymer materials (such as TPU, PEBA, and flexible photosensitive resin) are higher than those of standard PLA or ABS, especially for some medical-grade flexible materials, which are expensive. Moreover, in printing processes such as Fused Deposition Modeling (FDM), printing complex structures requires supports. The consumption of support materials and the subsequent removal process (especially for water-soluble supports) increase material and labor costs. Additionally, in Selective Laser Sintering (SLS) technology, although unsintered resin powders such as TPU can be partially recycled and reused, the material properties will deteriorate after multiple recyclings, requiring the addition of a large amount of new powder, which constitutes an implicit cost.

Flexible components fabricated through 3D printing are typically small in size, making it difficult to enhance printing efficiency. For instance, printing a moderately complex flexible part using DLP technology may take several hours or even days. This is because for flexible materials, excessively fast printing speeds can lead to issues such as poor interlayer adhesion, stringing, and poor surface quality, limiting their potential for speed increase. Moreover, the current 3D printing of flexible materials has strict requirements for environmental temperature and equipment stability, all of which increase printing costs.

## 4. Summary

In recent years, significant progress has been made in additive manufacturing technology within the field of flexible materials, particularly in material system development, process optimization, and functionalized applications. Current research focuses on the printable properties and functional modification of high-performance elastomers (e.g., thermoplastic polyurethanes TPU, silicone rubber), hydrogels, and photopolymer resins. By introducing nanoscale reinforcing phases (e.g., carbon nanotubes, graphene, or cellulose nanocrystals), the mechanical properties, conductivity, or self-healing characteristics of materials have been effectively enhanced. At the process level, technologies such as FDM, SLA, and DIW have enabled the fabrication of complex structures with micrometer-level precision. However, challenges remain regarding interlayer bonding strength, surface quality, and long-term stability. Notably, multi-material printing and gradient structure design have expanded emerging applications in flexible electronics and soft robotics. Nevertheless, current research faces challenges including the lack of comprehensive material–process–structure–performance correlation models and limitations in large-scale production. Overall, existing studies have established a solid foundation for customized fabrication of flexible devices, yet systematic solutions remain lacking for balancing material functional diversity, process reproducibility, and cost-effectiveness.

Given the limitations of current research, future work needs to advance in three dimensions: (i) material design, (ii) process innovation, and (iii) interdisciplinary integration. In terms of materials, efforts should focus on developing smart-responsive materials (such as pH/temperature/light-responsive hydrogels), biodegradable elastomers, and their composite material systems, while incorporating machine learning methods to accelerate material design and performance prediction. Material design proposes performance objectives, directly driving the innovation of printing processes; meanwhile, the breakthrough of process bottlenecks provides possibilities for realizing more complex material systems. Interdisciplinary integration serves as a link among the three, introducing cutting-edge concepts such as artificial intelligence and biomimetics, while subverting the design and process implementation paths of materials.

First, technologically, it is essential to advance multi-modal integrated printing systems that incorporate in situ monitoring and feedback control mechanisms, such as optical coherence tomography, to enable real-time process regulation and defect correction. Interdisciplinary collaboration with fields like biomedical engineering and electronic engineering should be further strengthened to foster breakthroughs in applications such as bionic organs and stretchable circuits. Additionally, establishing standardized performance testing protocols and databases, covering mechanical properties, fatigue life, and biocompatibility, is recommended to facilitate data sharing and comparative research. In addition, environmental sustainability is emphasized by developing low energy consumption printing processes and renewable materials.

Subsequently, 3D printing of flexible materials enables integrated “design-manufacturing” processes, particularly suitable for small-batch, high-complexity flexible component production (such as customized medical implants, flexible sensors, etc.), bringing breakthroughs to fields like robotics and wearable devices. More importantly, the integration of flexible material printing with digital technologies allows for precise process control and optimized resource utilization during manufacturing.

In conclusion, flexible composites fabricated by 3D printing still hold significant research potential and are rapidly evolving towards smart and functionally diverse directions to meet practical application requirements in fields such as encapsulation, soft robotics, bio-tissue engineering, and wearable monitoring. With the ongoing exploration of new material systems and novel printing technologies, this will inevitably drive the maturity and commercialization of flexible composites in the future. Finally, we hope this review will be helpful to researchers in this field.

The limitations of this paper:

Although this article systematically reviews the key challenges and deficiencies faced by 3D printed flexible materials at the material system and printing process levels, due to limitations in research scope and space, there are still several limitations in the following aspects, which are also important directions worthy of attention in future research:

The relationship between application scenarios and performance requirements needs further research. This article delves deeply into materials and processes, but fails to summarize the core requirements for materials and processes based on the performance requirements of specific application scenarios. Additionally, this article does not summarize the engineering challenges encountered during the transition from the laboratory to industrialization.

## Figures and Tables

**Table 1:** Common 3DP technologies used in flexible material manufacturing and their advantages and disadvantages.

3DP		Printing Accuracy	Material Type	Advantages	Disadvantages	Ref
Material extrusion (MEX)	DIW	100–250 μm	Silicone Rubber, Hydrogel, PU Resin, elastomer composite materials	User-friendly operation, strong design capabilities, complex multi-scale architecture, low cost	The preparation of printing inks with high rheology is required, which have low printing resolution and flow channels prone to clogging	[14,39,40,41,42,43,44]
	FDM		TPU (thermoplastic polyurethane elastomer), TPE (Thermoplastic Elastomer), Flexible PLA, Soft PLA	Multi-material structures, low-cost materials, complex structures	The printable material range is narrow, printing speed is fast, surfaces between layers are rough, and flow channels are prone to clogging	[45,46,47]
Vat photopoly-merization (VPP)	DLP	10–100 μm	Standard Flexible, Elastic Resin Rubber-Like Resin	High resolution, fast printing speed, large build volume, wide material range, high precision, and high accuracy	High process costs, material limitations, and limited availability of photocurable flexible materials	[48,49,50]
	SLA			Simple, fast manufacturing with high precision, capable of producing complex structures with numerous detailed features	Post-processing is required, the slurry requires high viscosity, and there are few photo-curable flexible materials available	[51,52]
Other	PBF	50–200 μm	TPUpowder, Thermoplastic polyamide elastomer, Blended Powders	Low cost, no need for supporting materials	Inhalation risk, rough surfaces requiring complex post-processing, messy powder residue, and high costs	[53,54,55]
	BJT	50–150 μm	TPU Flexible polyurethane resin, nylon powder, flexible adhesive	High production efficiency, no supporting structures required, and relatively low cost	The anisotropy of the sample is significant, limiting material selection	[56,57,58]
Multi-technology	SLA/ other	25–500 μm	PDMS	Complex processes, require different processes tailored to various materials	3D printing has the capability to manufacture complex shapes and enable customization, while conventional or other technologies provide high resolution, material properties, or functional characteristics	[59]
	UV/ DIW	5–580 μm	Silicone Rubber			[60,61,62,63]
	FDM/electrospinning	----	PCL			[64]

**Table 2:** Types of flexible materials for 3D printing.

Material Type		3D Printing	Application Scenarios	Advantages	Disadvantages	Ref
Thermoplastic flexible materials	TPU, TPS SBS, TPC TPA, PEBA	FDM FFF SLS	Functional components, footwear and apparel, soft robots, industrial parts	High elasticity and flexibility; High wear resistance and fatigue resistance; Strong functionality and good overall resilience; Diverse wire options	Poor long-term creep resistance; Limited high temperature resistance; The difficulty of printing is high; Yilasi, precision control is difficult; Supporting is extremely difficult to handle; Poor surface finish	[145,146,150,159,162,163]
Light-cured flexible resin material	PUA, PEGDA UV-PDMS, PC, Silicone Resin, NBR, epoxy resin, PI, etc.	DLP SLA	Medical models, precision components	Extremely high printing accuracy; The printing speed is fast; No need to deal with mechanical feeding issues	Insufficient durability; Poor long-term stability, prone to aging; The post-processing procedure is cumbersome and poses hygiene risks; The material cost is relatively high	[168,169,170,171,172,173,174]
Hydrogel-based flexible materials	gelatin, alginate, hyaluronic acid, PEGDA, GelMA Composite hydrogels	DLP SLA DIW	Tissue engineering, drug delivery, soft actuators, biosensors	Excellent biocompatibility; Responsiveness to external stimuli; High light transmittance; Material exchange capacity;	The tear resistance and toughness are very poor; Poor structural stability; It is difficult to control liquidity; Poor long-term stability	[181,182,183,184,185,186,187,188,189]
Flexible composites	Elastomer-based composites, hydrogel composites, light-cured flexible resin composites	DLP SLA DIW FDM	Flexible electronics, bionic structures, intelligent robots, and intelligent protection	Significant enhancement of mechanical properties; Realize functionalization and intelligence; Improve dimensional stability; Multi-material application;	The difficulty of printing has increased; It is difficult to ensure the uniformity of materials; Sacrificing some flexibility and elasticity; The surface quality may deteriorate; Limited material system	[197,198,199,200,201,202,203,204,205,206,207,208,209,210,211,212,213,214,215,216,217,218,219,220,221]

## Data Availability

No new data were created or analyzed in this study. Data sharing is not applicable to this article.

## Abbreviations

The list of abbreviations

FDM	Fused Deposition Modeling
DLP	Digital Light Processing
TPE	Thermoplastic elastomers
VPP	Vat Polymerization
BJT	Binder Jetting
PLA	Poly Lactic Acid
PCL	Polycaprolactone
HME	Hot-melt extrusion
LCDs	Liquid crystal displays
DMD	Digital micromirror devices
BCC	Body-centered cubic
TPA	Thermoplastic polyamide elastomers
PEGDA	Polyethylene glycol
PNIPAM	poly(N-isopropylacrylamide)
NAM	N-isopropylacrylamide
HEMA	Hydroxyethyl methacrylate
DES	Diethyl siloxane
NFC	Near-field communication
CNTs	Carbon nanotubes
FFF	Fused Filament Fabrication
PPY	Polypyrrole
SLA	Stereolithography
SLS	Selective Laser Sintering
TPU	Thermoplastic polyurethane
PBF	Powder Bed Fusion
MEX	Material Extrusion
PDMS	Polydimethylsiloxane
ABS	Acrylonitrile Butadiene Styrene
CNFs	Cellulose nanofibrils
SIFS	Solidification
MWCNT	Multi-walled carbon nanotubes
TPC	Thermoplastic copolyester
PEBA	Polyether Block Amide
GelMA	Methylacryloylated gelatin
CNC	Cellulose nanocrystals
TFEA	2,2,2-trifluoroethyl acrylate
AA	Acrylic acid
AFE	Automatic fiber embedding
HA	Hydroxyapatite
UA	Acrylic urethane
IPNs	Interpenetrating polymer networks
CNN	Convolutional Neural Network
